# From Protein Film Electrochemistry to Nanoconfined Enzyme Cascades and the Electrochemical Leaf

**DOI:** 10.1021/acs.chemrev.2c00397

**Published:** 2022-12-27

**Authors:** Fraser A. Armstrong, Beichen Cheng, Ryan A. Herold, Clare F. Megarity, Bhavin Siritanaratkul

**Affiliations:** † Department of Chemistry, University of Oxford, South Parks Road, Oxford OX1 3QR, United Kingdom; ‡ Stephenson Institute for Renewable Energy and the Department of Chemistry, University of Liverpool, Liverpool L69 7ZF, United Kingdom

## Abstract

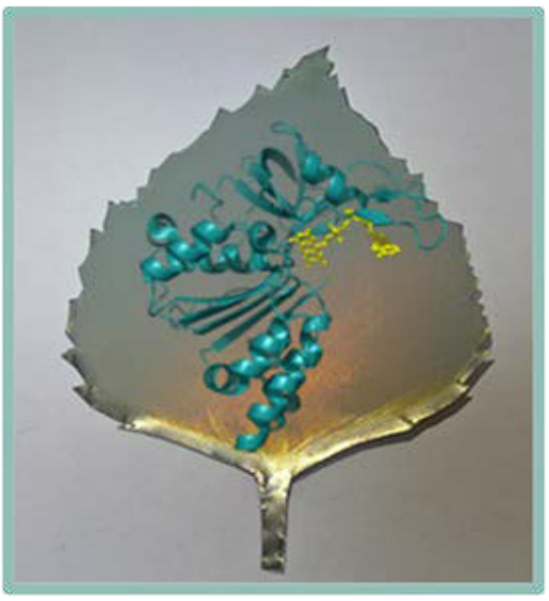

Protein film electrochemistry (PFE) has given unrivalled insight into the properties of redox proteins and many electron-transferring enzymes, allowing investigations of otherwise ill-defined or intractable topics such as unstable Fe–S centers and the catalytic bias of enzymes. Many enzymes have been established to be reversible electrocatalysts when attached to an electrode, and further investigations have revealed how unusual dependences of catalytic rates on electrode potential have stark similarities with electronics. A special case, the reversible electrochemistry of a photosynthetic enzyme, ferredoxin-NADP^+^ reductase (FNR), loaded at very high concentrations in the 3D nanopores of a conducting metal oxide layer, is leading to a new technology that brings PFE to myriad enzymes of other classes, the activities of which become controlled by the primary electron exchange. This extension is possible because FNR-based recycling of NADP(H) can be coupled to a dehydrogenase, and thence to other enzymes linked in tandem by the tight channelling of cofactors and intermediates within the nanopores of the material. The earlier interpretations of catalytic wave-shapes and various analogies with electronics are thus extended to initiate a field perhaps aptly named “cascade-tronics”, in which the flow of reactions along an enzyme cascade is monitored and controlled through an electrochemical analyzer. Unlike in photosynthesis where FNR transduces electron transfer and hydride transfer through the unidirectional recycling of NADPH, the “electrochemical leaf” (e-Leaf) can be used to drive reactions in both oxidizing and reducing directions. The e-Leaf offers a natural way to study how enzymes are affected by nanoconfinement and crowding, mimicking the physical conditions under which enzyme cascades operate in living cells. The reactions of the trapped enzymes, often at very high local concentration, are thus studied electrochemically, exploiting the potential domain to control rates and direction and the current–rate analogy to derive kinetic data. Localized NADP(H) recycling is very efficient, resulting in very high cofactor turnover numbers and new opportunities for controlling and exploiting biocatalysis.

## 1 Introduction

Living organisms need to secure and use energy in efficient ways. Some familiar phenomena are electrical in nature and directly amenable to physical chemistry equations, examples being ion transport across membranes or electron transport between membrane-bound complexes catalyzing photosynthesis or respiration: in these cases, it is usually not difficult to predict whether energy is released or consumed in a particular step. In contrast, the reactions of complex organic molecules, by far the most diverse processes occurring in a cell, are less tractable thermodynamically; for example, it is not obvious why (or the degree to which) one metabolite is more stable than another or why an extended sequence of steps in a network of reactions will proceed in a certain direction. In addition, the catalysts that perform these reactions are usually crowded in enclosed environments, a far step from the homogeneous state of a dilute solution that has long been the foundation of enzyme kinetics. Electrochemistry, the field that links electricity and chemistry, offers important ways to unify and unravel the links between the fundamental thermodynamics and obscured kinetics that control the efficient use of energy in living organisms.

Redox reactions relevant for biology have been studied by electrochemical methods for well over half a century. For characterizing protein-bound redox cofactors, the early focus was on static methods, i.e., potentiometry, in which small electron mediators were used to facilitate equilibration in spectrophotometrically monitored redox titrations. The objects were to determine “midpoint potentials” for generating a thermodynamic scale for bioenergetics, rationalize the properties of different types of redox centers according to their oxidizing or reducing power, and detect otherwise unseen centers through the spectroscopic signatures of particular oxidation states. Before the 1970s, it was widely assumed that no electrode would allow fast and direct electron exchange with a molecule as large as a protein.^1^

*Dynamic* electrochemical methods are those in which electrical current, directly related to the rate of a reaction, is measured as a function of electrode potential or time. Techniques like cyclic voltammetry and chronoamperometry have long been standard laboratory procedures for chemists investigating the electron-transfer reactions of coordination complexes and a range of different electrocatalysts, but they are less familiar in laboratories specializing in enzymology or biocatalysis (even though the earliest recognition of electrical phenomena stemmed from observations made with organisms).

During the 1970s, it began to be realized that proteins much larger than 10 kDa in size could exhibit direct, quasireversible diffusion-controlled cyclic voltammetry at certain electrodes: the breakthrough came when two groups independently published results showing the quasireversible electrochemistry of cytochrome c, a mitochondrial electron-transfer protein.^1−3^ It was also noted that carbon electrodes coated with hydrogenase equilibrated rapidly with the 2H^+^/H_2_ redox couple at the reversible potential.^4^ The field of protein dynamic electrochemistry that emerged has become well established, with new experiments yielding a wealth of mechanistic information that could not have been obtained by other means. Particular attention has been directed at investigating proteins that are anchored to (adsorbed upon) an electrode, thus requiring minuscule amounts of material and avoiding the masking of information due to slow protein diffusion. The approach became popularly known as protein film electrochemistry (PFE).^5−13^ An important advantage of dynamic electrochemical methods is that they measure how the rate of a reaction depends on the thermodynamic driving force—the electrode potential being varied continuously, as in voltammetry, or held constant while the time dependence is monitored (chronoamperometry). The current gives a direct readout of the overall rate at any given potential—information in the “potential domain”: PFE has thus proved to be a valuable new way to characterize the catalytic properties of redox enzymes.

A common feature of enzymes displaying *direct* electrocatalysis (i.e., without requiring a small electron mediator) is that they contain redox-active centers positioned sufficiently close to the protein surface to allow direct electron tunnelling to/from the electrode. These centers may be long-range electron-relay sites, or the site of catalysis itself. Many enzymes fall into this category, particularly those for which the biological role involves transfer of electrons to/from a second protein partner or the quinone pool contained within a membrane. The electron-relay system operating within an enzyme should always be regarded as an inherent part of the catalytic machinery: this is because the properties of every component may contribute to determine the catalytic rate and bias the direction of electron flow. Enzymes containing a redox-active site that is not used for long-range electron transfer are not naturally equipped to be electrocatalysts; hence the reason that detection of their activity has long relied on small mediators that diffuse rapidly and are able to access buried active sites that are equipped instead for innershell reactions with small molecules like oxygen. Mediators can be covalently attached to an enzyme to form a “wire”, and particular use of such procedures has been made in the biosensor field with subjects like glucose oxidase: they may also be attached to the electrode surface in a conducting polymer.^14^ However, use of a mediator masks valuable information; most obviously, the direction and rate become controlled by the properties of that mediator (the reduction potential) rather than any inherent feature of the enzyme.^1^

**Figure 1:**
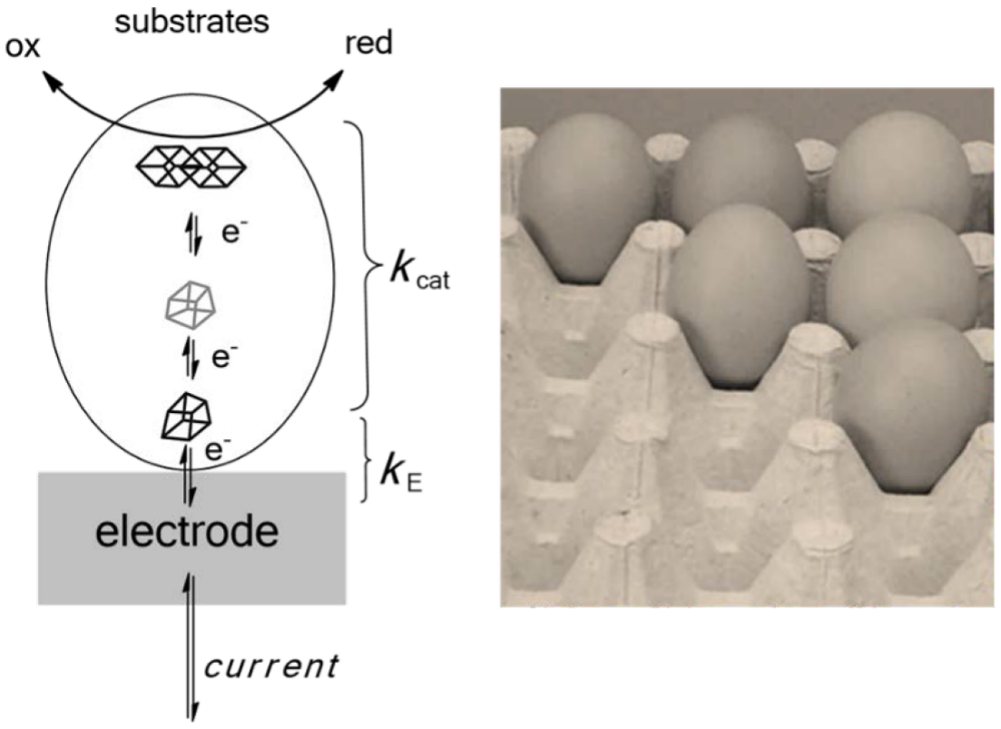
Concept of protein film electrochemistry (PFE). (left) An enzyme is immobilized on an electrode surface in such a manner that a short-range electron tunnelling route operates between the electrode surface and an electron entry/exit site that terminates a relay to/from the active site. The enzyme retains full activity with regard to reactants diffusing from solution. (right) A rough electrode surface greatly increases the probability that the enzyme will bind tightly and make a good electron-transfer contact.

A central feature of PFE is that the enzyme is confined to an electrode surface at which its reactions are activated by electron tunnelling (Figure 1, left).

Later in this Review, we describe how PFE is extended to a nanoporous electrode, thereby allowing the investigation and exploitation of enzymes loaded into the material. We show how extended catalytic cascades that include enzymes lacking any direct redox role can be brought under tight electrochemical control. We start by summarizing how PFE has played a unique role in unravelling some fundamental characteristics of biological electron transfer and catalysis. During the development of PFE, the importance of attaching the enzyme to an electrode surface soon became clear, as immobilization affords sharply defined voltammograms, rapid response times, minimal sample requirements, and the ability to add and remove reactants and inhibitors simply by exchanging the buffer. Even on a flat surface, the adsorbed enzymes are in a concentrated state of nanoconfinement, in this case “2D”, but nonetheless in an environment that may be closer to that experienced in an organelle or membrane than the dilute solution that is normally used in enzyme kinetics.

**Figure 2:**
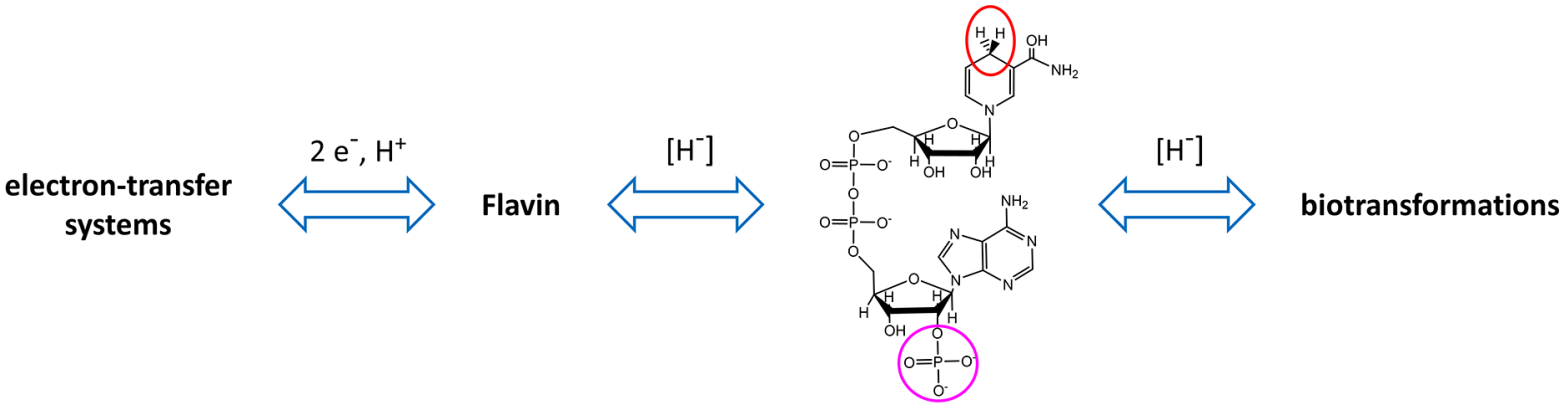
Position of nicotinamide cofactors in relation to biological oxidation and reduction processes. An enzyme-bound flavin group for which an intermediate radical state is sufficiently stable can translate between long-range one-electron transfer and covalent hydride transfer. For most redox biotransformations, the hydride entity is transferred in a highly selective fashion by enzymes using NAD(P)^+^/NAD(P)H cofactors. Circles indicate positions distinguishing NAD(P)^+^ and NAD(P)H (top) and NAD(H) and NADP(H) (bottom).

The major part of the Review is then devoted to the theme of nanoconfinement and the wider advantages that it offers to enzymes, first in situations found in living cells (membranes, organelles) and then in recent work intended to mimic the biological containment of enzymes through artificial scaffolds or enclosures. We then discuss how 3D nanoconfinement is now being used to study extended enzyme cascades by electrochemical techniques, making possible wider applications in biocatalysis and tight coupling to myriad dehydrogenases and nonredox enzymes. Crucial steps forward are the rapid and reversible electrochemistry of nicotinamide cofactors, NAD(P)(H), molecules that had evaded the efforts of electrochemists since their discovery a century ago, and the ability to channel cascade catalysis within a nanoporous electrode material. Most of the redox chemistry in a cell is carried out by nicotinamide cofactors which are vehicles for transferring the “hydride” entity (a 2e^–^, 1 H^+^ package) between enzymes, and there may be great advantages in channelling such transfers to ensure the cofactors are rapidly recycled (Figure 2).

## 2 Protein Film Electrochemistry

Protein film electrochemistry is described in several reviews: these have explained the principles, provided numerous examples, including applications, and stressed the importance of attaching an enzyme directly to the electrode surface, thereby avoiding sluggish protein diffusion that masks useful information.^5−13^ Protein molecules adsorb spontaneously on many electrode surfaces, often with assistance from polyvalent counterions. The assumption is made that the electrode is 2D, although not flat—surface roughness allowing multiple enzyme–electrode interactions and making orientation less critical for electron tunnelling. A good analogy is an egg: placed on a flat table, an egg will roll around with minimal surface contact, but placed in an egg tray (Figure 1 right), there is a high probability that a proficient tunnelling pathway, broadly exponential in distance dependence, will be engaged. Atomically “flat” electrodes need to be modified with functionalities to tether the enzyme at a suitable site. The importance of polar and hydrogen bonding interactions between enzyme and electrode has recently been emphasized.^15^

### 2.1 Electron Exchange with Active Sites

**Figure 3:**
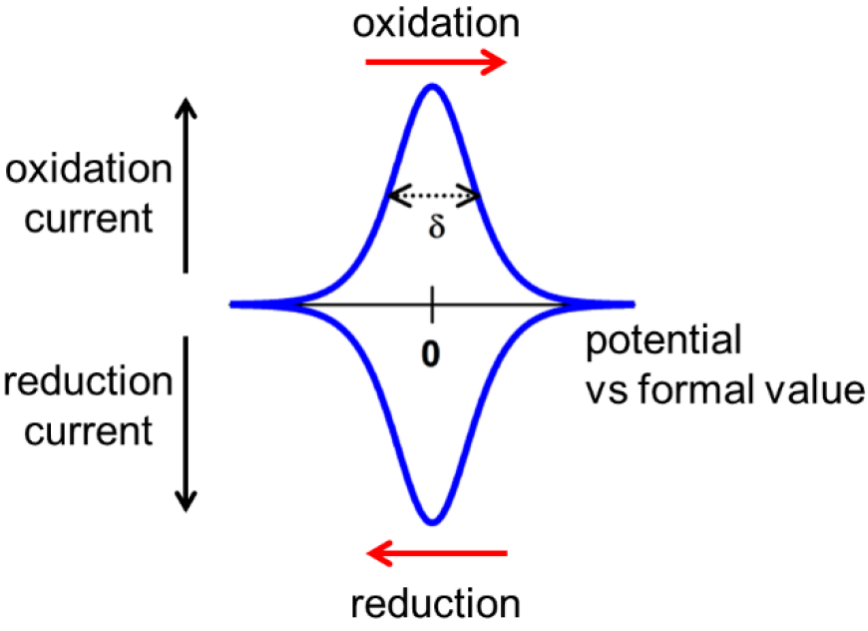
Parameters of importance extracted from a “nonturnover” signal (the background current has been removed). (1) The average peak potential = formal potential: for a reversible electron-transfer reaction, the two peaks will coincide. (2) If *n* is the number of electrons transferred in an ideal cooperative manner, the peak height varies as *n*^2^, and the half-height width varies as δ = 90/*n* mV at room temperature. Consequently, a cooperative two-electron process (where a one-electron intermediate is extremely unstable) will give a peak current 4× that of a one-electron reaction, and δ = 45 mV for both oxidation and reduction peaks. As the one-electron intermediate in a two-electron process becomes more stable, δ increases (the signal ultimately dividing into two). (3) Inhomogeneity among the redox sites (commonly known as dispersion) broadens the peaks. (4) The area under each peak corresponds to the charge passed, and the number of sites can be calculated from charge/*nF*, where *F* is the Faraday constant.

Once immobilized, the coverage of a protein may be sufficiently high that electron exchanges between the electrode and active sites, as driven during cyclic voltammetry, give rise to visible peaklike signals that are finite in size and not distorted by a diffusive tail (Figure 3). Léger and co-workers have developed a very useful program for processing PFE results: it includes the analysis of these signals, which are often faint and lie above a large capacitance background.^16^

The theory for cyclic voltammetry of immobilized (diffusionless) redox couples as developed by Laviron and others leads to several clear and valuable predictions.^17^ First, the width at half height should be 90/n mV at room temperature (*n* is the number of electrons transferred in the step), so a fully cooperative two-electron reaction should have an ideal width of approximately 45 mV in both oxidative and reductive directions. Second, the height (amplitude) should depend on *n*^2^, so the peak current of a signal due to fully cooperative two-electron transfer may be 4 times higher than that of a one-electron reaction. The narrow, intensified signals of two-electron transfers make them easier to detect above a large capacitance background. Third, the area enclosed by an oxidation or reduction peak can be used to determine the electroactive concentration of the redox-active species on the electrode surface, simply by using the Faraday equivalence and knowing the number of electrons transferred. Fourth, the formal reduction potential, obtained from the average of oxidation and reduction peaks, should be close to that of the redox couple measured in solution provided the protein has not undergone a structural change at the electrode surface: this condition is more likely to be met for a large molecule where the active site is well shielded by the outer shell (while retaining efficient electron tunnelling). Fifth, dispersion (environmental inhomogeneity) will broaden the peaks. Sixth, the kinetics of electron transfer and coupled processes such as the rate-limiting binding of a proton or ligand can be studied by measuring how the positions of peaks shift as the scan rate is varied. These aspects dominated much of the field during the 1990s, particularly in applications to [Fe–S] centers in small proteins.^18−21^

### 2.2 Enzymes as Direct Electrocatalysts

Despite the clear guidelines for their analysis and exploitation for investigating small electron-transfer proteins, observations of such peak-type cyclic voltammograms for an enzyme in the absence of a substrate (nonturnover signals) are rare. Enzymes are usually too large to give a sufficiently high coverage on the surface of an electrode, 2 pmol/cm^2^ being a reasonable lower practical limit for detection of signals due to a one-electron transfer. In the rare cases where nonturnover signals are observed, addition of the appropriate reactant converts the peaks into a catalytic wave, and the turnover frequency of the enzyme at any potential can be calculated by dividing the catalytic current by the electroactive coverage. If high scan rates can be used, it is also possible to estimate the turnover frequency from the point at which nonturnover peaks emerge in a “trumpet plot”.^19,20,22^ Mainly, however, the goal of PFE when applied to enzymes is to measure electrocatalytic activity that stems from otherwise “undetectable” active sites. Depending on the turnover rate and enzyme coverage, catalytic waveforms may be limited by (i) reactant depletion (leading to a peaklike waveform); (ii) the inherent activity of the enzyme, i.e., the turnover frequency (leading to a sigmoidal waveform); or (iii) interfacial electron transfer (producing an extended linear appearance to the waveform, modified by dispersion effects).^23^ Transport of reactants to the electrode surface (or products away from the electrode) can be controlled by rotating the electrode at speeds over several thousand rpm.

**Figure 4:**
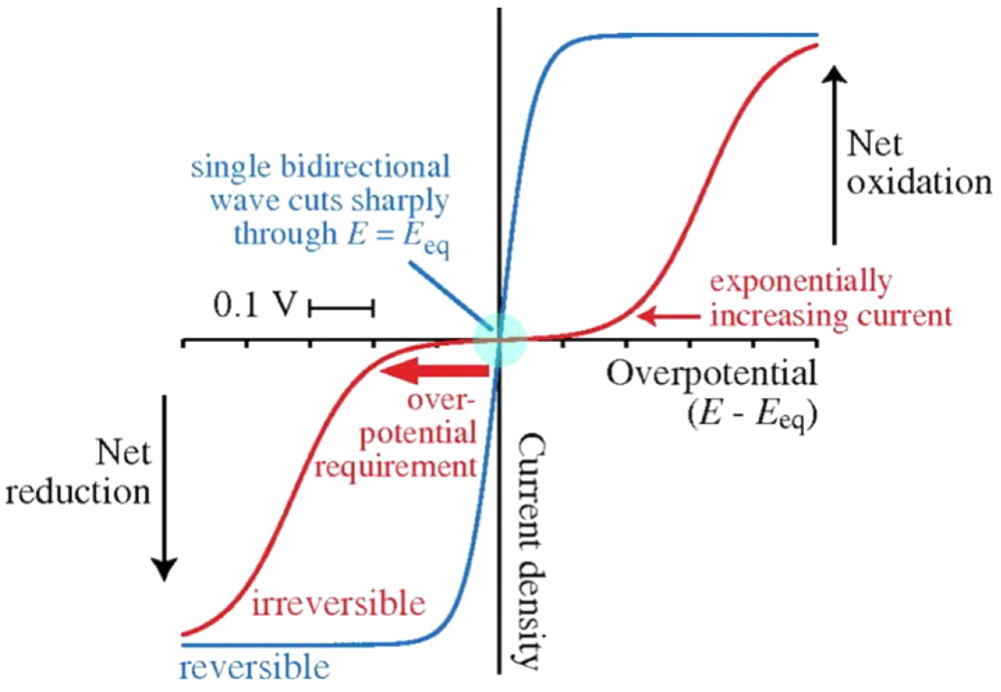
Distinguishing reversible and irreversible electrocatalysis. Steady-state electrochemical kinetics visualized by cyclic voltammetry when both oxidized and reduced forms of a redox-active species are present (in this case at equal concentrations, so the equilibrium potential *E*_eq_ approximates to the formal potential for the redox couple). A reversible electrochemical reaction (one with a large exchange current density) produces a single sigmoidal wave (blue) which cuts (without inflection) through the zero-current axis at *E*_eq_ (green circle) and ultimately reaches a potential-independent limiting current in either direction at relatively low overpotential. Conversely, if the exchange current density is very low, the current is negligible near the formal potential, and two separate sigmoidal waves are observed (red) for oxidation and reduction. The waves emerge from the baseline with an exponential dependence on potential. A substantial overpotential is required to match the current produced by the reversible system. (Adapted with permission from ref (24). Copyright 2011 PNAS.)

Many enzymes have now been established to be reversible electrocatalysts when attached to an electrode.^24^ Reversible electrocatalysis is defined here as a situation in which only a minimal overpotential is required to drive a reaction in each direction, and the rate at potentials close to the reversible value roughly follows that derived, ultimately, from the Nernst equation. Thus, if a mixture of oxidized and reduced forms of a reactant are present in solution, the current cuts sharply through the potential axis at the expected potential. A comparison between reversible and irreversible electrocatalysis is shown in Figure 4.^24^

**Figure 5:**
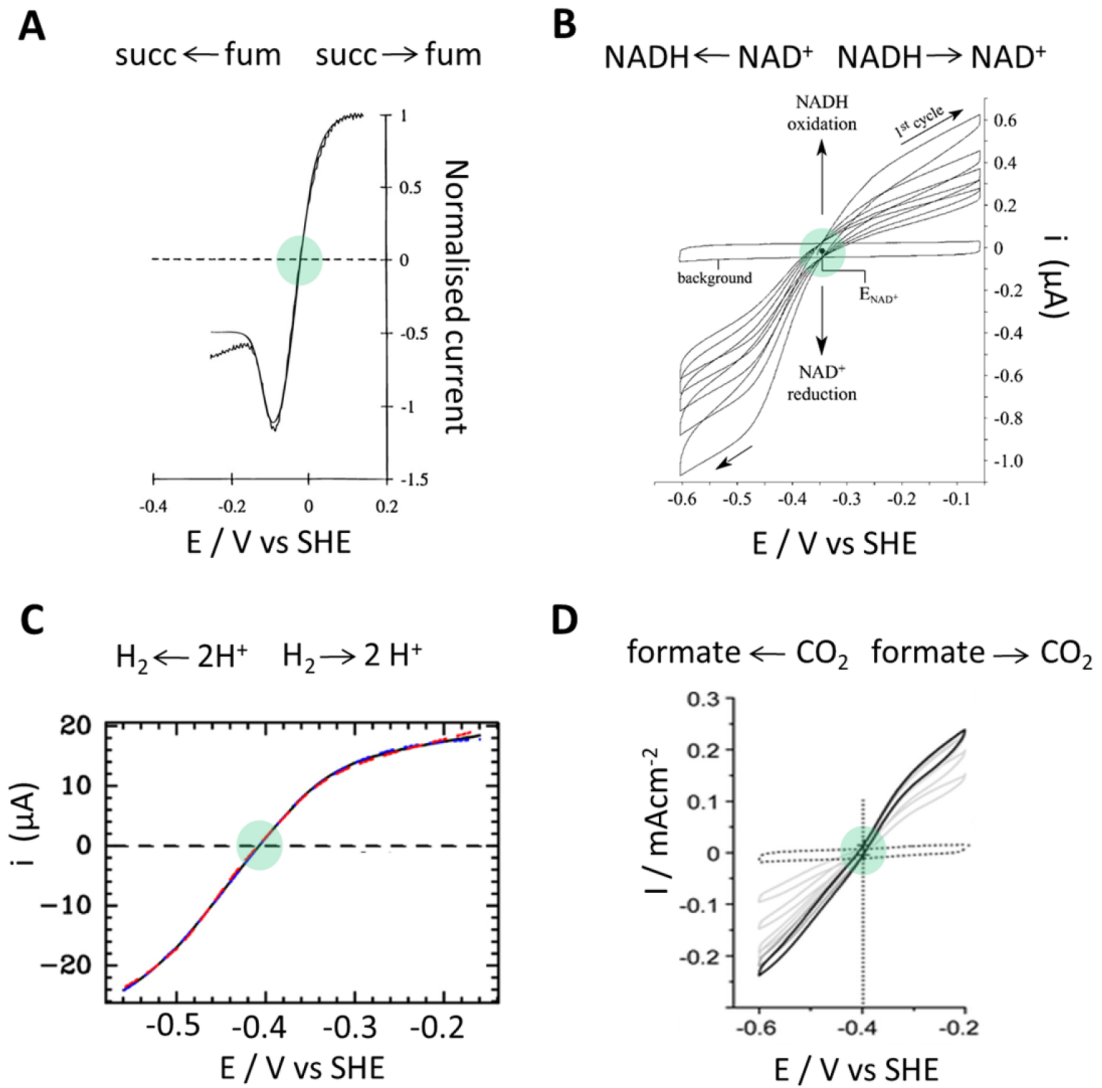
Examples of reversible electrocatalysis by enzymes, highlighting in each case (green circle) the formal potential measured for specific conditions. In cases where the capacitance current is high and the electrocatalysis is unstable, the formal potential is traced out as an isosbestic point in successive voltammograms. (A) Succinate dehydrogenase, measured with a 50:50 fumarate/succinate mixture. (Adapted with permission from ref (28). Copyright 1996 American Chemical Society.) (B) Truncated form of Complex 1. (Adapted with permission from ref (29). Copyright 2003 American Chemical Society.) (C) [FeFe] hydrogenase experiment overlaid on a model. (Adapted with permission from ref (30). Copyright 2021 American Chemical Society.) (D) Formate dehydrogenase. (Adapted with permission from ref (31). Copyright 2014 American Chemical Society.)

Among established chemicals and materials, only metals of the platinum group are known to display reversible electrocatalysis, in this case referring to the 2H^+^/H_2_ redox interconversion that forms the basis of many fuel cell and electrolysis technologies. Recent advances have been made in discovering metal complexes that catalyze 2H^+^/H_2_ redox interconversion, and there has been progress with other important reactions such as CO_2_ reduction to CO or formate.^25^ A major issue for simple catalysts is how to combat the “scaling relation” that correlates the energies of major species along the reaction coordinate: for instance, where the advantageous stabilization of the transition state is offset by the undesired stabilization of the bound product.^26^ In contrast, enzymes excel at H_2_ or CO_2_ activation, and they are now established as reversible electrocatalysts for many more complex reactions, a fact that suggests the general importance of overpotential minimization during early evolution—i.e., biology could not afford to waste energy. Some examples are shown in Figure 5. Using impedance spectroscopy, it has been possible to estimate electrocatalytic “exchange” rate constants at the reversible potential, and values of 78 and 12 mol (2H^+^/H_2_) s^–1^ for two [FeFe]-hydrogenases confirm how inherently active these enzymes are without any driving force at all.^27^

**Figure 6:**
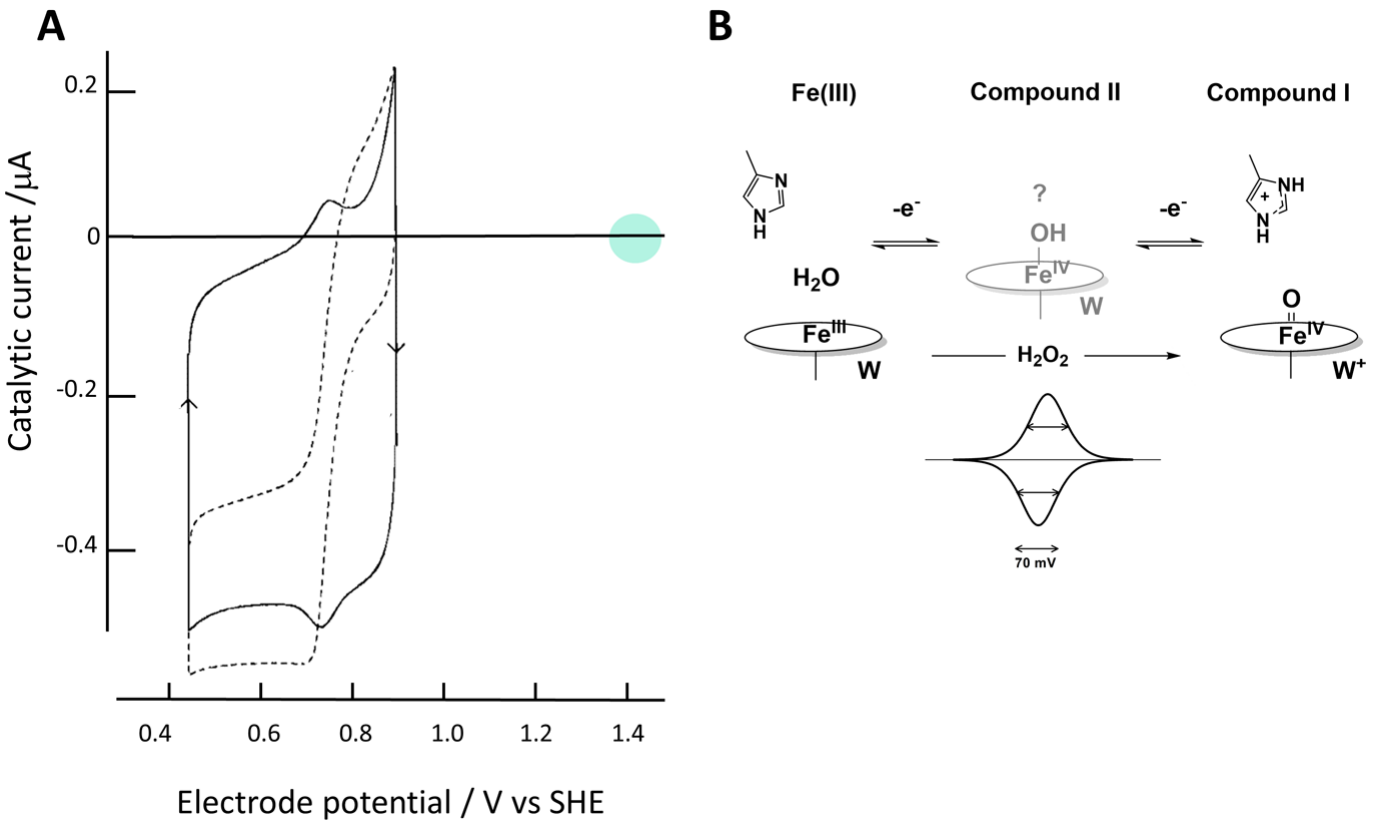
The redox chemistry of yeast cytochrome c peroxidase: (A) as revealed by protein film electrochemistry; (B) as interpreted in terms of active site chemistry. (A) (Solid line) cyclic voltammogram obtained after allowing the enzyme to adsorb from dilute solution at pH 6.1 on a pyrolytic graphite edge electrode. The dashed line (corresponding to current axis) was recorded after adding H_2_O_2_ to give a 20 μM concentration. The green circle indicates the formal reduction potential (+1.41 V) for H_2_O_2_ at pH 6.1, demonstrating that electrocatalytic reduction is highly irreversible. (B) The sharp, symmetrical nonturnover peaks centered at +0.74 V vs SHE with δ = 70 mV correspond to a cooperative two-electron process, with the one-electron intermediate (Compound II) slightly unstable with respect to Fe(III) and Compound I, which consists of Fe(IV) (ferryl) and a tryptophan radical. (Adapted with permission from ref (32). Copyright 1996 American Chemical Society.)

The stabilization of the critical inner- and outershell structure made possible by the large size of enzymes compared to surface atomic or small molecular electrocatalysts was an advantage, not a disadvantage—one that could be honed by mutation and selection during evolution.^7^ A question that often arises is how much of the macromolecule might be “chipped away” while still retaining catalytic activity. For an enzyme to be a reversible electrocatalyst, it is important that the thermodynamics associated with the catalyst (electron/proton transfer coupled to reactant bonding and product release in a single cycle) match those of the overall reaction being catalyzed. Enzyme electrocatalysis may be irreversible even when a reversible nonturnover signal is observed. An example of the latter is cytochrome c peroxidase (Figure 6) for which the reduction potential for the Compound I/Fe(III) couple, observed clearly as a cooperative two-electron signal in PFE experiments, is approximately +0.74 V vs SHE at pH 6, far more negative than that for the H_2_O_2_/2H_2_O couple (+1.4 V).^32^ In traditional redox potentiometric titrations, in which a small-molecule mediator is added to allow equilibration of an active site with the electrode potential, the best mediator is usually one having a reduction potential close to that of the active site of the protein. Reversible electrocatalytic behavior also depends on tight coupling between electron and proton transfers, a simplistic view being that the overall transfer into a low dielectric medium becomes electrically neutral, thus lowering the Coulombic barrier. A recent study of two [FeFe]-hydrogenases, in which a proton-transfer (PT) pathway lying diametrically opposite to the electron-transfer pathway is marginally altered (replacing glutamate by aspartate) to delay PT, resulted in the introduction of small overpotentials for both H_2_ oxidation and H_2_ evolution.^33^ Although catalytic cycles are often assumed to apply to both directions, it is important to note that microscopic reversibility in electrochemistry applies only when a specific potential is defined: different mechanisms can be used for oxidation and reduction that occur under very different conditions of driving force.

Catalytic bias refers to the inherent tendency of an enzyme to catalyze a reaction in a preferred direction, i.e., oxidation or reduction. The effect on reversible electrocatalysis is to cause the current responses observed either side of the reversible (zero-current) potential to differ, and we may refer to Figure 5. Assuming that rates are not limited by reactant depletion, and both reactant and product are present at equal concentrations in solution, an enzyme having zero catalytic bias may display an electrocatalytic wave that is roughly symmetrical in shape about the zero-current potential (highlighted as a green circle), as shown earlier in Figure 4. If instead, the enzyme is strongly biased to act in the oxidation direction, the rise in oxidation current will be much stronger than that observed for the reduction current, but the wave will still cross the potential axis at the reversible value.

Noting first of all that a catalyst cannot alter the position of equilibrium, the rules for catalytic bias are straightforward, at least at the basic level. Three factors are important, the first being the thermodynamic compatibility between the enzyme and the reaction being catalyzed: this is expressed as the difference between the reduction potential of the reaction and the potential at which electrons enter or leave the catalyst. A large difference produces a massive catalytic bias in one direction, the irreversible electrocatalytic reduction of H_2_O_2_ by cytochrome c peroxidase (Figure 6) being a good example. The site in the enzyme that is responsible for determining the potential is called the electrochemical control site or center (ECS):^34−36^ it may be the actual site of catalysis or an electron-relay center, particularly one that receives or donates electrons to an external reaction partner. The second (and related) influence on catalytic bias is the inhibitory consequence of a product that is formed in one direction being very tightly bound. Both of these direct influences on catalysis have been recognized for over a century, originating from the work of Sabatier.^37^

The third factor relates to the status of the enzyme at any specific potential: here, as an extreme example, the active site may undergo a secondary reaction that results in inactivation. In electrocatalysis, a familiar case is the “passivation” of a metal by formation of an oxide coating, which prevents the metal catalyzing an oxidation reaction. In enzyme electrocatalysis, such behavior is noted for hydrogenases, where the application of an oxidizing potential, intended to increase the rate of H_2_ oxidation, results instead in the formation of an oxidized metal–OH adduct, and the activity decreases.^34^ The electrochemical detection of such potential-optima effects by PFE was first noted for succinate dehydrogenase, for which a region of negative resistance is displayed just below the reversible potential (Figure 5A):^28^ this property, readily detected by PFE, instigated the notion that electron-transferring enzymes resemble the components of electronic circuits. More complex models are thus needed to account for wave-shapes and catalytic bias at a detailed level, as enzymes rarely conform to the simple limiting cases shown in Figure 4. In many cases, catalysis is limited by electron transfer, giving rise to a persistent slope, as more driving force is needed to meet the demands of the active site.^23^ Léger and co-workers have developed a more detailed model that takes into consideration the rates and energetics of intramolecular electron transfer, and Figure 5C shows how well experimental data for a [FeFe]-hydrogenase can be modeled.^30^

The direct connection between catalytic current and rate facilitates studies of the kinetics of activation or inactivation processes where, normally, a concentration vs time dependence would be analyzed to extract the rate of change of rate, i.e., the double derivative. Examples include hydrogenases, where the formation of inactive resting states and their reductive reactivation are easily studied by chronoamperometry. The reactions are thus monitored directly—the current decreasing or increasing when an inhibitor or activator is injected, or when the potential is stepped to initiate or stall a process. The ability to impose strict potential control on all the enzyme molecules of a sample allows certain steps of a complex reaction pathway to be blocked and then restarted—a tactic used to examine the final stages of assembly of the active site “H-cluster” of [FeFe]-hydrogenases.^38^ As with other types of heterogeneous catalysis, solution-based reagents can be replaced whenever required.

### 2.3 Summary of the Advantages of PFE

The established advantage of PFE is that it adds the potential dimension to enzyme catalysis. Enzymes display characteristic voltammetric electrocatalytic wave-shapes, which may be regarded as signatures. Although it is usually difficult to measure absolute rates, relative rates (ox vs red) and activation potentials are easily derived and represent unique information. Direct electrochemical control also makes it possible to interrupt important chemical steps, such as the final assembly of the H-cluster mentioned above.

The electrodes used for PFE, for example pyrolytic graphite ‘edge’ (PGE) are usually regarded as providing a 2D environment. The surface concentration of electroactive enzyme may be sufficiently high that reactant is depleted close to the electrode surface, yet steady-state conditions are still achieved by using a rotating disc electrode to ensure a constant flux of incoming reactant and assistance for the outgoing product. The electrochemical leaf (the e-Leaf) described later takes PFE a very large step further, since the electroactive enzyme is buried within a porous electrode and acts on an exchangeable cofactor for immediate use by other enzymes, located nearby, that are *not* electroactive. The advantage of adding nanoconfinement to PFE does not stem from any increase in concentration of a *single* enzyme: it arises from the gathering together and mutual confinement of two (or more) different enzymes that are sequential partners along a cascade, conferring the ability to retain exchangeable cofactors and channel intermediates within a catalytic network. As we describe in the next section, these are conditions that exist in living cells.

## 3 Nanoconfinement in Biology

In expanding PFE to include nanoconfined enzyme cascades, it is instructive to understand how cooperative catalysis and channelling occur in living cells. Unlike industrial processes carried out in large-volume reactors, biology uses trillions of nanoreactors connected by energy- and information-transfer networks to achieve higher rates and efficiency. The complexity of biomolecular systems such as energy metabolism resulted, through evolution, in high levels of efficiency and control, organized at the levels of both pathway and coordinated interplay.^39^ Metabolism involves the perpetual flow and ebb of complex networks of chemical reactions that drive the flow of energy and matter. Its finely tuned organization is achieved by *compartmentalization* based on temporal (time-dependent) and chemical confinement, spatial confinement, or a combination of these principles. In this section, we identify some lessons from biology that can be taken forward for *bioinspired* tandem catalysis and electrocatalysis.

### 3.1 Temporal and Chemical Confinement

Temporal confinement (the ability to switch pathways on and off, or to control flux when required) coordinates central metabolism with the cell cycle.^40−42^ Metabolic pathways must function in concert and respond to control signals; their activity is thus modulated at different times by differential transcription and control of specific cascade enzymes by allosteric/signaling molecules or by substrate availability. In budding yeast, surges in respiration exhibit periodicity, coordinated with the expression of over half its genome.^43^ Reactive oxygen species, redox and Ca^2+^ signaling,^44^ and cyclic AMP^45^ are all under spatiotemporal control.^46−48^

Chemical compartmentalization, achieved by using different cofactors for the same reactions, allows metabolic pathways to run without competition. The most obvious exploitation of this strategy occurs in the liver where NAD(H) and NADP(H) are central to the compartmentalization of metabolic processes and in muscle cells where phosphate is transported by the inert energy carrier, phosphocreatine.^49,50^

### 3.2 Spatial Confinement—The Advantages for Biology

**Figure 7:**
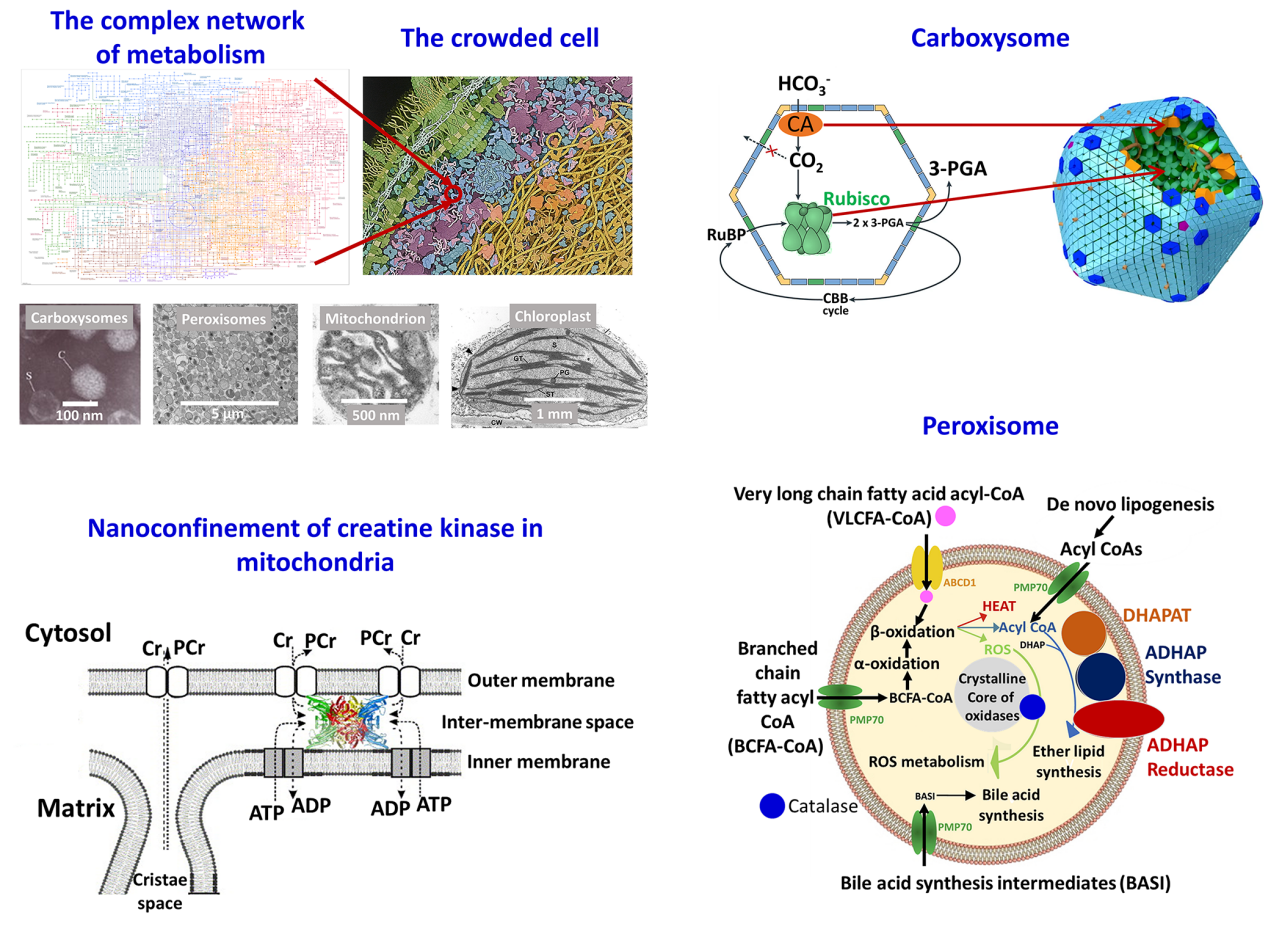
Nanoconfinement in Biology. (top left) The intricate network of metabolism (Kegg^57^ map 01100 Metabolic Pathways) functions under confinement in the crowded cell (illustration by David S. Goodsell, RCSB Protein Data Bank. DOI: 10.2210/rcsb_pdb/goodsell-gallery-028) and inside organelles such as the mitochondrion,^58^ the chloroplast,^59^ carboxysomes,^60^ and peroxisomes.^61^ (top right) The carboxysome. The left image shows a cartoon of the bacterial microcompartment with entrapped RuBisCO and carbonic anhydrase and the flow of substrates and products. (Adapted with permission from ref (54). Copyright 2018 Springer Nature.) The right image shows the arranged polypeptide icosahedral shell structure from *Cyanobium* PCC7001 packed with RuBisCO and carbonic anhydrase; the shell prevents loss of CO_2_ to the cytoplasm. (Adapted with permission from ref (62). Copyright 2018 Springer Nature.) (bottom left) Octameric creatine kinase wedged in the intermembrane space of the mitochondrion. (Adapted with permission from ref (63). Copyright 2006 Elsevier.) (bottom right) The peroxisome with its main metabolic pathways shown, including β-oxidation of very long chain fatty acids, α-oxidation of branched chain fatty acids, synthesis of bile acids and ether-linked phospholipids, and removal of reactive oxygen species. The peroxisomes in some types of cells also contain a dense crystalline core of oxidative enzymes, shown here in gray. (Adapted with permission from ref (64). Copyright 2014 Elsevier.)

Most relevant to the focus of this Review is the spatial nanoconfinement achieved by harboring enzymes and metabolites inside enclosures—membrane-divided vesicles such as mitochondria, lysosomes, vacuoles, chloroplasts,^51^ or protein-cages such as bacterial microcompartments (BMCs).^52−56^ Some examples are shown in Figure 7.

Nanoconfinement may also be based on restricting intracellular diffusion, as with septins in membranes,^65,66^ and liquid–liquid phase separation^67^ allows for compartmentalization without organelle encapsulation.^68−71^ The metabolic pathways within each compartment rely on external signals such as Ca^2+^ influx, energy in the form of ATP, and specific metabolite precursors and cofactors; thus, communication between the pathways must also be achievable.^51^ The many benefits of spatially nanoconfining cascades include prevention of unwanted metabolic “crosstalk”, protection of the cell from toxic intermediates, the ability to tailor internal conditions, attainment of high local enzyme concentrations,^62^ control of the selective entry of reactants, and the entrapment of exchangeable cofactors and intermediates, many of which may be unstable.

Spatial confinement allows a cell to achieve selective control over cascades that utilize the same enzymes for different purposes, for example, the catabolic and anabolic pathways that connect pyruvate and glucose. In each pathway there must be at least one reaction not common to both, which is highly unfavorable in the reverse direction. Futile interconversions of metabolites by opposing catabolic and anabolic pathways are thus prevented by their physical segregation; for example, the pathway for fatty acid catabolism is confined to mitochondria whereas the counterpart synthetic pathway operates in the cytosol. Interference in cell signaling is avoided by recruiting signaling protein complexes to lipid rafts in the cell membrane, thus providing an isolated compartment where they are concentrated and where their specific phosphorylation state is controlled to prevent interference with downstream signaling.^72,73^

The use of different isozymes confined in separate locations facilitates the fine control evident in the metabolism of muscle cells which is tailored for *in situ* production of ATP on demand. The creatine phosphate shuttle maintains homeostasis by exploiting spatial and temporal confinement to maintain a constant pool of ATP.^50^ Key to this task is creatine kinase which catalyzes the production of ATP from phosphocreatine;^50^ it exists as compartment-specific isoforms: a dimer in the cytosol and an octamer (at high concentration) in the mitochondrial intermembrane space.^74,75^ Compartmentalization allows each isozyme to carry out a specific function: the mitochondrial version is localized close to processes that produce ATP whereas the cytosolic enzyme is functionally coupled to processes that both require ATP (e.g., ion pumps) and use ATP.^76^ As an inert energy carrier, phosphocreatine links sites of high ATP production to ones having high ATP demand;^77^ unlike ATP, it is metabolically inactive, and its lower charge and smaller size allow it to diffuse more rapidly.^76^ The octamer is squeezed into the intermembrane space (9–15 nm) where it complexes with a porin and a translocator, both of which directly channel substrate and product to and from the enzyme, facilitating exclusive production of phosphocreatine.^63^ The cytosolic dimer associates with myosin-binding protein-C, providing ATP to nearby ATPases.^78,79^ The system’s efficiency relies on channelling, concentration in mitochondrial spaces (∼0.01 mM octamer^80^), confinement of the cytosolic dimer in a different zone, and chemical compartmentalization of phosphocreatine.

Physical confinement also allows cascades to operate under bespoke conditions; for example, the synthesis of extremely hydrophobic lipids occurs in association with the membrane of the smooth endoplasmic reticulum rather than in the aqueous cytoplasm.^81^ Confinement also limits the damaging effects that intermediates of one pathway may have on others; for instance, confinement of the central enzymes of oxidative metabolism in mitochondria minimizes oxidant-induced mutation of nuclear DNA and interference with redox signaling.^82^ By confining oxidative pathways to the peroxisome, the release of hydrogen peroxide in the cytoplasm is prevented, and its decomposition is facilitated by coentrapment of catalase under optimal alkaline conditions.^83^ Confinement inside peroxisomes extends to other pathways, including the oxidation of fatty acids and the synthesis of bile acids and ether-linked phospholipids.

A classic example of biology’s use of nanoconfinement to enhance cascade efficiency occurs in cyanobacterial carboxysomes where the quantities and ratios of entrapped enzymes are optimized.^60^ These microcompartments have diameters of 100–400 nm with walls 3–4 nm thick; most of their volume is taken up (60–70% of the entire particle mass) by two enzymes—carbonic anhydrase (CA) and ribulose-1,5-bisphosphate carboxylase (RuBisCO), the primary photosynthetic carbon-fixing enzyme.^84,85^ Their concentration ratio is optimized to meet needs: for example, in *Halothiobacillus neapolitanus*, the ratio of CA/RuBisCO is approximately 1:7,^86^ compensating for the inherently slow rate of RuBisCO. Carboxysomes are vital for the cyanobacterial carbon-concentrating mechanism because CO_2_ is produced *in situ* for RuBisCO by the action of carbonic anhydrase, while photorespiration is curtailed by creating a barrier to O_2_. The strategy is so effective that McGrath and Long^87^ predicted that incorporation of the cyanobacterial carbon-concentrating mechanism into terrestrial C_3_ crops such as rice and wheat could increase yields by 36–60%.

In addition to the carboxysome, cascades in bacteria are entrapped in other BMCs^65,88^ to perform functions such as propanediol utilization.^52−56^ Segregation in prokaryotes is also achieved by organelles with a lipid bilayer such as photosynthetic membranes,^89^ the internal membrane structures of the *Planctomycetes* and the magnetosomes of magnetotactic bacteria.^90^

#### 3.2.1 Crowding of Enzymes in Biological Compartments

The cytoplasm and the internal spaces of organelles are highly crowded with macromolecular concentrations reaching levels as high as 400 g L^–1^ in mitochondria^91^ and 200 g L^–1^ in the cytosol.^92^ The term “crowding” is used to describe these environments rather than “highly concentrated” because no singular macromolecule is necessarily at high concentration (see section 5.3). Enzymes and their pathways have evolved to function in this crowded state, which is far removed from conventional laboratory conditions.^92−95^ Macromolecular crowding in the cytoplasm contributes to a form of encapsulation by phase separation, affecting the partitioning of ions,^96^ protein–protein association,^97^ and separation of products.^96,98^ Many assumptions used in conventional enzyme kinetics break down for crowded enzyme environments.^99^ In most studies on the effect of crowding on enzyme kinetics, the Michaelis–Menten constant is decreased by crowding whereas the effects on *k*_cat_ vary, increasing for some enzymes^100^ and decreasing for others.^101^ Crowding retards the diffusion of small solute molecules^102^ but enhances binding rates.^93^ Cells may even exploit crowding to direct the diffusion of molecules toward specific zones and away from others.^103^

#### 3.2.2 Nanoconfinement in Multisubunit Complexes and Metabolons

**Figure 8:**
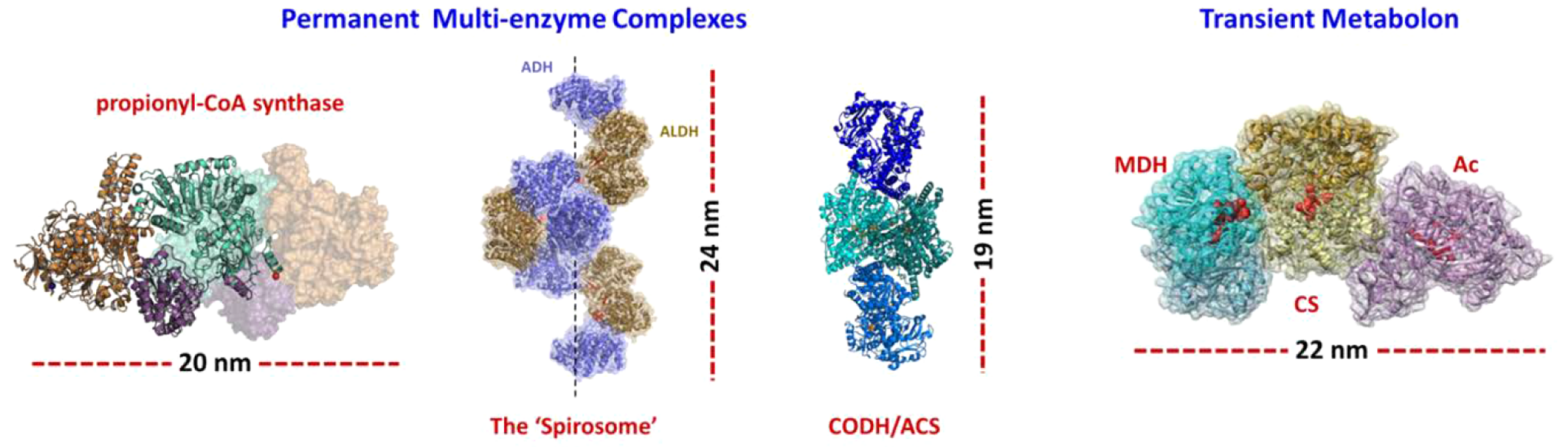
Examples of permanent and transient multienzyme complexes, with their dimensions. From left to right: Propionyl-CoA synthase from *Erythrobacter sp*. NAP1. (Adapted with permission from ref (105). Copyright 2018 Springer Nature.) The “spirosome”, a permanent complex of alcohol dehydrogenase and aldehyde dehydrogenase that protects the bacterial cell from release of acetaldehyde. (Adapted with permission from ref (106). Copyright 2020 Springer Nature.) Carbon monoxide dehydrogenase/acetyl Co-A synthase from *Moorella thermoacetica* (PDB: 1MJG); model of the putative metabolon formed by the association of malate dehydrogenase (MDH), citrate synthase (CS), and aconitase (Ac). (Adapted with permission from ref (107). Copyright 2015 John Wiley & Sons.)

Multienzyme complexes, both permanent and transient, minimize or prevent the undesired escape of intermediates along a specific pathway.^104^ Permanent multienzyme complexes form at transcription, and their domains do not dissociate, whereas transient complexes, known as metabolons, form in response to certain conditions. Some multienzyme complexes are shown in Figure 8.

### 3.3 Substrate Channelling

#### 3.3.1 Substrate Channelling in Permanent Multisubunit Enzyme Complexes

Preventing the loss of intermediates is most reliably achieved if the enzyme’s domains are formed from a single polypeptide chain^108^ rather than dissociable domains with weak interactions at domain interfaces.^109,110^ This aspect is exploited in the production of engineered chimeras for biosynthesis.^111,112^ The polyketide synthases (PKSs) and the nonribosomal peptide synthetases (NRPSs) are multidomain enzymes likened to “factory assembly lines” with modular architecture. The individual catalytic domains in these huge structures work cooperatively, with intermediates channelled in a defined order; the growing polymeric product is never lost to the surroundings.^113^

Fatty acid synthases (FASs) catalyze iterative 2-C additions; the complexes consist of six enzyme units and one acyl carrier protein (ACP).^114^ Active sites are aligned to receive the acyl intermediates (covalently attached to the ACP) by a swinging arm mechanism.^115^ This mechanism is also employed in electron transfer in the 2-oxoglutarate dehydrogenase complex and in enzymes with prosthetic groups such as biotin, for example, pyruvate carboxylase:^116,117^ the same strategy has been exploited *in vitro*.^118^ The tryptophan synthase complex^119^ is constructed so as to prevent loss of an indole intermediate, which undergoes conformationally triggered channelling through a 25 Å long tunnel to the final catalytic site.^119−121^ Likewise, a 30 Å tunnel in guanosine monophosphate synthetase prevents escape of the ammonia intermediate.^122^ For a comprehensive review on channelling by multidomain enzymes, see Raushel et al., 2003.^123^

Conformational changes and intersubunit communication also drive substrate access and transfer in propionyl-CoA synthase (Figure 8), an enzyme that catalyzes a three-step reaction during CO_2_ assimilation.^105,124^ The enzyme exists both as a complex formed by three separate enzymes^125^ and as a single polypeptide with three catalytic domains,^124^ suggesting that these options evolved independently of each other, with the naturally fused version optimized for channelling of the highly reactive and toxic intermediate, acrylyl-CoA.^105^ The three catalytic sites face into an enclosed space [inner diameter ∼3.5–5.5 nm (∼33 nm^3^)] lined with positive residues to promote CoA-ester retention; any small gaps in its walls are surrounded by negative charges to repel the negatively charged intermediates, preventing their escape.

Aldehyde-alcohol dehydrogenases (AdhE) are permanent assemblies consisting of ADH and ALDH molecules bound together by short amino acid residue linkers, resulting in an extended dynamic structure known as a spirosome (Figure 8).^106,126^ They are mainly found in bacteria and some unicellular eukaryotes and are responsible for converting acetyl-CoA to ethanol. Upon binding of the NADH cofactor, the spirosome undergoes a conformational change to its “extended” form, creating a channel between the ADH and ALDH active sites, thereby avoiding release of the toxic intermediate, acetaldehyde, into the cell.^106^

Synthesis of coenzyme A in some anaerobes is carried out by a bifunctional enzyme formed by tight association between two Ni-containing components, carbon monoxide dehydrogenase (CODH) and acetyl CoA synthase (Figure 8). Carbon monoxide produced from CO_2_ at the active site of CODH (the C-center) is channelled through a tunnel 140 Å in length for incorporation into acetyl-CoA at the CH_3_-binding A-cluster on the ACS domain: an essential but toxic intermediate is thus retained.^127^

#### 3.3.2 Substrate Channelling by Transient Assemblies of Enzyme Complexes

Metabolons and metabolic channelling were first noted for the mitochondrial and other central pathways.^128,129^ Metabolons assemble under temporal control, in response to conditions such as substrate concentration or stage of cell cycle.^130^ An intuitive view of these transient complexes, which lack a membrane or other constraint, is that of a relay in which the enzymes are arranged close together and in sequence, intermediates being passed from active site to active site. Benkovic defined a metabolon as “*a dynamic enzyme complex carrying out the sequential steps of a metabolic pathway by cluster channelling, where an intermediate can be processed by any of the multiple copies of each enzyme instead of depending on the nearest one*.”^130^

Channelling of intermediates is observed for the tricarboxylic acid cycle enzymes malate dehydrogenase (MDH) and citrate synthase (CS).^131^ In the complex shown in Figure 8, which includes a molecule of aconitase (the next enzyme along the cycle), the active sites of MDH and CS are approximately 6 nm apart.^132^ Brownian dynamics simulations^133^ and experimental data^134^ suggest that the oxaloacetate intermediate is electrostatically guided between the two sites.

The transient nature of metabolons makes them difficult to study directly. In 1985, the tricarboxylic acid cycle became the first example of an experimentally verified metabolon;^135^ since then, metabolon assembly has also been proposed for glycolytic enzymes that colocalize to the outer mitochondrial surface ensuring a supply of pyruvate for respiration,^136,137^ and in the chemotactic assembly of the first four enzymes of glycolysis under crowded levels similar to those found in the cell.^138^ The enzymes of the Calvin–Benson–Bassham pathway localized in the chloroplast stroma have been isolated as complexes from pea^139,140^ and spinach,^141,142^ and the structure of an *Arabidopsis thaliana* complex consisting of glyceraldehyde-3-phosphate dehydrogenase, chloroplast protein (CP12), and phosphoribulokinase has been solved.^143^ Seminal experiments involving fusion proteins in *Arabidopsis thaliana*, transient expression in *Sorghum bicolor*, and metabolon isolation and reconstitution in liposomes, demonstrated the dynamic nature of a metabolon for dhurrin synthesis.^144,145^

The most persuasive work on metabolons involves *de novo* purine biosynthesis,^146^ in which 5-ribosyl-1-pyrophosphate (PRPP) is converted in ten steps to inosine monophosphate (IMP) which is then used to make AMP or GMP. The ten steps are catalyzed by a six-enzyme metabolon called the purinosome, the transient assembly of which correlates with cellular purine requirement.^147−149^ Highlights of a recent review^146^ include the mechanism by which the metabolon is transported to its target^150^ and direct visualization of the metabolon in action.^151^

Evidence for substrate channelling *in vivo*, and whether/how it confers a kinetic advantage in metabolic flux, is an ongoing debate.^152,153^ Channelling has been verified in the glycolylic^137,154^ and dhurrin^144,145^ metabolons and in the purinosome^151^ but is yet to be established for the Calvin–Benson–Bassham cycle.^155^ Direct channelling of NADH between glycerol-3-phosphate dehydrogenase (GDH) and l-lactate dehydrogenase (LDH) has been hypothesized^156^ and contested.^157^ The scope for such localized cofactor recycling is revisited in section 5.8. Sweetlove and co-workers showed that channelling by glycolytic enzymes results in an increase in flux.^154^ Regarding the *extent* to which channelling occurs and whether it actually confers a kinetic advantage,^158^ the authors suggested that channelling should enhance kinetic flux only if the rate of the nonchannelled pathway was limited by diffusion of intermediates. For most metabolites, diffusion is very fast compared to enzyme turnover—collisions between enzymes and substrates being 1000–10000 times faster than the typical specificity constants for the enzymes in central metabolism.^159^ The two scenarios for which diffusion would limit and therefore for which channelling may have significant effects are (1) if the enzymes of the pathway are so dilute that the average separation distance is large enough for diffusion to become limiting (though this situation cannot apply to metabolons) and (2) if an enzyme exhibits such high efficiency that the rate is limited by diffusion. In section 4, we outline some artificially confined cascades and studies to test the benefits of substrate channelling.

### 3.4 Relevance of Biological Nanoconfinement for Electrocatalysis

As will be discussed in section 5, a large proportion of electrochemical cascade catalysis by the e-Leaf is bioinspired, not by structurally mimicking enzyme active sites (the traditional view) but by using a thin nanoporous electrode material to crowd and encapsulate enzymes in optimal ratios, thereby making possible highly efficient cascade (tandem) reactions in which intermediates are channelled rather than dispersed. The question will ultimately arise as to whether it is feasible to design and build networks based on simple (small molecular) catalysts into nanoconfined electrode materials that approach the sophistication levels we have summarized for biological systems.

## 4 Nanoconfining and Energizing Enzyme Cascades for Technology

Artificially confined enzyme cascades are of considerable interest for biocatalysis technology and fundamental insight, and they have been extensively reviewed.^160−168^ In this section, we highlight some recent approaches to advance the design of scaffolds and interpretation of data.

### 4.1 Artificial Confined Cascade Systems

**Figure 9:**
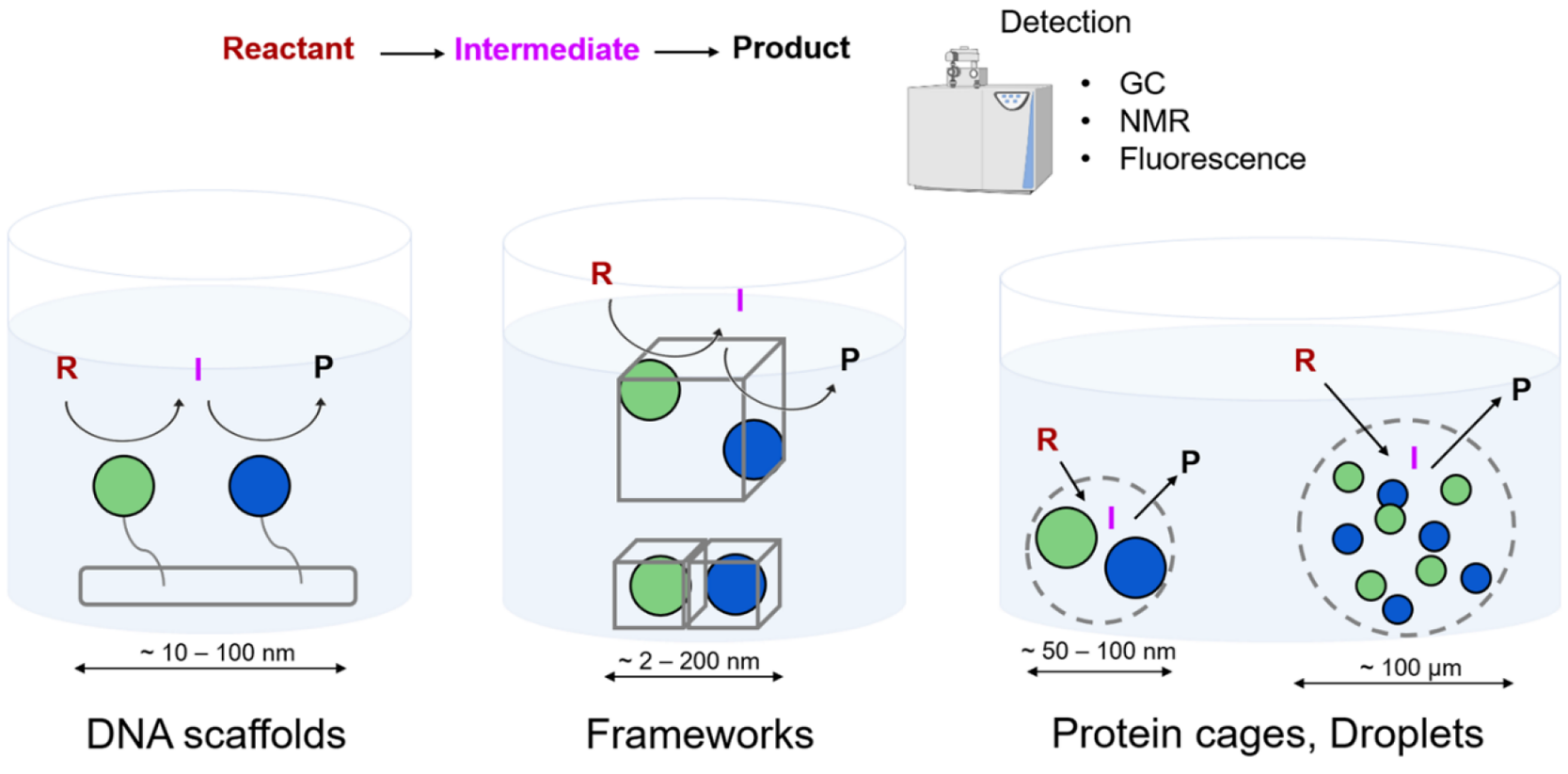
Diagrams of artificial enzyme cascades with their typical length scales. From left to right: DNA scaffolds, metal–organic and covalent–organic frameworks, and encapsulation by protein cages or droplets.

Artificial confinement of enzyme cascades can be broadly categorized into immobilization/tethering and encapsulation/compartmentalization (akin to the “surface-confined” and “volume-confined” in ref (160)), while recognizing that there may be overlapping cases. The types and dimensions of these “open” and “closed” systems are shown in Figure 9.

#### 4.1.1 DNA Scaffolds, Swinging Arms

Scaffolding enzymes to DNA is the most widely reported procedure for spatially organizing enzymes in cascades at the nm level. The DNA nanostructures can be designed to have free strands, or tethers, to which DNA-conjugated enzymes can be bound.^169−171^ Kahn et al. used an octahedron DNA scaffold to investigate, systematically, the effects of spacing and orientation of each enzyme in a 2-enzyme cascade and found that coimmobilizing both enzymes increased the cascade rate compared to the freely diffusing control. However, the effects of different distances on the scaffold were small, indicating that the rate enhancement probably has greater contribution from local environment effects, rather than proximity channelling. Other DNA nanodevices have been reported,^172−175^ including dynamic scaffolds.^176^

In the case of the intermediate being a cofactor that is recycled between two enzymes (i.e., an exchangeable cofactor), this entity can also be tethered to a scaffold to yield a “swinging arm” motif,^177^ with the aim of restricting the free diffusion of the cofactor and enhancing its recycling efficiency. In one example, a modified NAD(H) cofactor was immobilized on a DNA scaffold between a glucose-6-phosphate dehydrogenase and a malic dehydrogenase, enzymes that catalyze NAD^+^ reduction and NADH oxidation, respectively. The range over which the NAD(H) could diffuse was determined by the cross-linker used (in this case disuccinimidyl suberate, DSS, 11.4 Å). The activity of the cascade was increased by 2 orders of magnitude compared to the case of freely diffusing cofactor. This concept has been extended to a 2D DNA scaffold^178^ and longer linkers.^179^

#### 4.1.2 Metal–Organic and Covalent–Organic Frameworks

Metal–organic frameworks (MOFs) and covalent–organic frameworks (COFs) can provide well-defined pore structures for accommodating enzyme cascades. Many studies on MOFs and COFs have focused on the encapsulation of single enzymes for enhanced stability, especially under harsh conditions.^180,181^ Our focus is on multienzyme catalysis, and we highlight studies that have incorporated more than one type of enzyme to incorporate a cascade into a rigid structure. Such encapsulated cascades are normally constructed by mixing the components in solution and allowing the framework to self-assemble around the enzymes. Pore size is a major consideration: cavity diameters range from <2 nm in “micropores” to 50 nm in “mesopores”, although pores in hierarchical systems can be as large as 200 nm. Many examples of multienzymes@MOF systems have been reported—for example, ZIF-8 (formed from Zn^2+^ and 2-methylimidazole) has been used to encapsulate the much tested model system for enzyme cascades, i.e., glucose oxidase (GOx) and horseradish peroxidase (HRP). Frameworks have also been used to support cascades composed of metal nanoparticles (NPs) and enzymes:^182,183^ for example, glucose oxidase was coupled to NiPd NPs (which acted similarly to a peroxidase) on a MOF.

#### 4.1.3 Protein Cages

The protein shells (capsids) of viruses provide a method for generating precise compartments for enzyme cascades.^184−186^ In one example, the shell of bacteriophage P22 was loaded with a glucose dehydrogenase for NADPH recycling (using glucose as the reducing substrate) and a NADPH-dependent carbonyl reductase.^186^ A fusion protein of both enzymes plus the scaffold protein of P22 was created and then self-assembled with the coating protein to yield a 65 nm diameter compartment containing, on average, 29 glucose dehydrogenase and 176 carbonyl reductase molecules (at concentrations of 0.89 and 5.0 mM, respectively).^186^ The encapsulated cascade could operate with [NADPH] as low as 1 μM, and fluorescence measurements indicated that the P22 shell was permeable to NADP(H).

#### 4.1.4 Droplet Encapsulation

Well-defined water-in-oil droplets (92 μm in diameter) have been used to encapsulate isolated thylakoid membranes capable of light-driven NADPH and ATP regeneration.^187^ Erb and co-workers showed that it is possible to use these droplets to produce artificial photosynthetic systems by incorporating the appropriate enzymes. Extensive investigations were carried out to evaluate the performance of an encapsulated 16-enzyme system devised to synthesize glycolate from CO_2_ via a crotonyl-CoA carboxylase/reductase cascade.^187^

#### 4.1.5 Enzyme Cascades on Particles

Coimmobilizing enzymes on particles that can be suspended in solution to drive a cascade reaction provides some of the benefits of fast solution kinetics while also retaining one of the main advantages of heterogeneous catalysis—the ability to separate the catalyst (magnetically or by centrifugation) and reuse it after the reaction is finished. An alcohol dehydrogenase and formate dehydrogenase were coadsorbed on agarose beads on which NAD(H) was also immobilized in such a way that it could be recycled between the two enzymes. The microbeads were used to carry out catalytic reduction of prochiral ketones by formate without any requirement for NAD(H) in bulk solution.^188^

Advantages are also gained by using particles that are electronically conducting. An early study described a catalytic system in which a hydrogenase and a second electroactive enzyme were adsorbed on particles of graphite produced by grinding up a material successfully used in PFE.^189^ Electron conduction through the graphite thus allowed reduction reactions at the second enzyme (nitrate reductase or fumarate reductase) to be driven using H_2_ gas oxidized at hydrogenase.^189^ Using an electroactive NAD^+^-dehydrogenase as the second enzyme, the concept was extended by Reeve, Vincent, and co-workers to allow the use of H_2_ to recycle NADH for biocatalysis.^190,191^

### 4.2 Characteristics of Confined Enzyme Cascades

In general, the aim of confining a cascade of enzymes, whether by immobilization onto scaffolds or encapsulated in compartments, is to increase the overall reaction rate. Although it might be expected that holding elements of a cascade in close proximity would lead to enhanced rates, this does not occur in all cases, and this section outlines the current understanding of the kinetics of confined enzyme cascades.

#### 4.2.1 Kinetics of “Open” Scaffolded Cascades

**Scheme 1:**
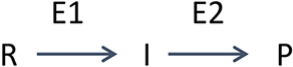
Linear Cascade

For a cascade assembled on a scaffold that allows free diffusion of intermediates, Kondrat^192,193^ and Hess,^194−196^ among others, have argued that substrate channelling by proximity alone does not increase the steady-state rate of product formation. In most studies, the simplest model considered (Scheme 1) is a linear cascade, catalyzing the conversion of the primary reactant R to product P via intermediate I, and comprised of just two enzymes. Here and elsewhere, E1 is the first enzyme of a cascade, E2 the second, and so on.

**Figure 10:**
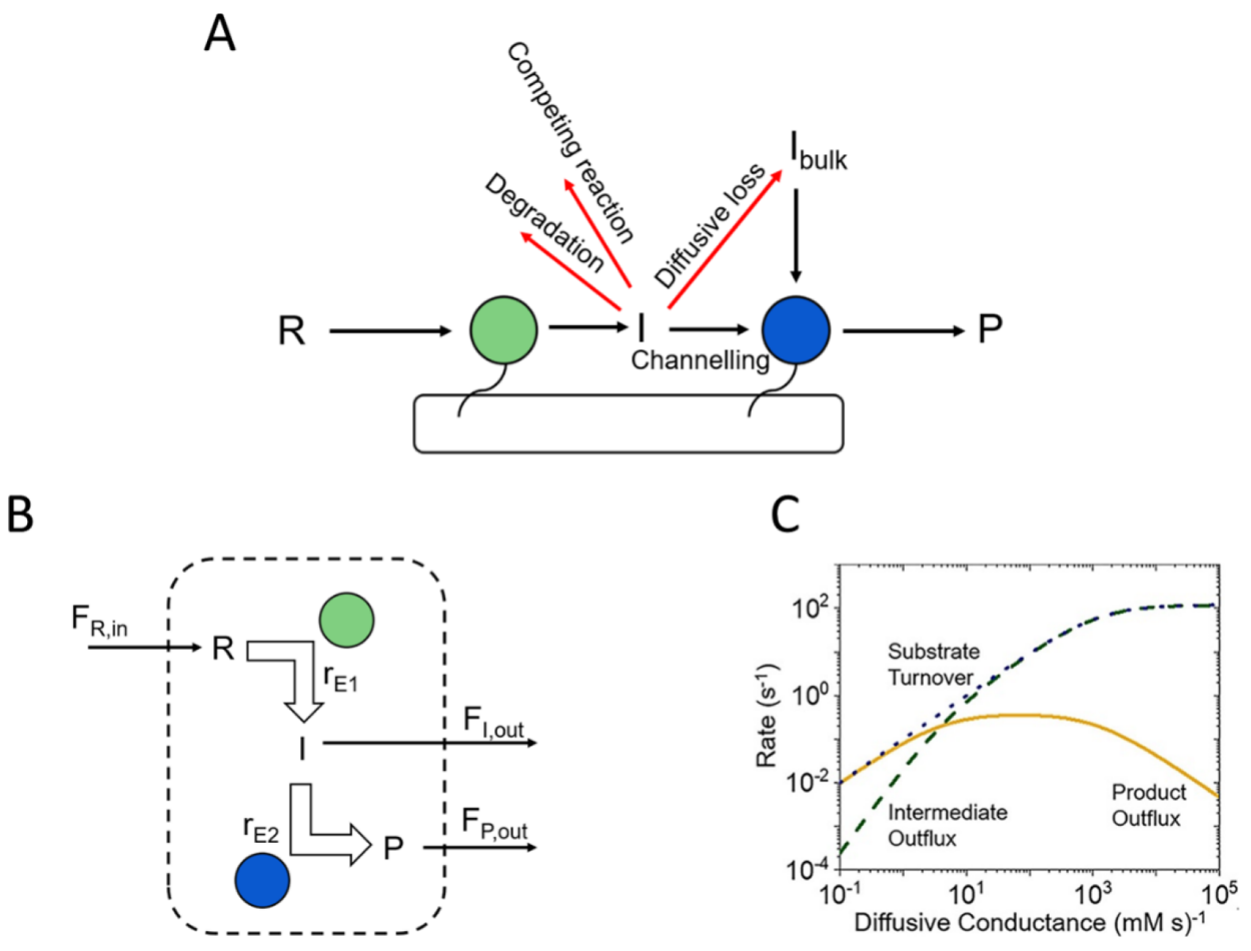
(A) Diagram of various pathways for an intermediate of a scaffolded cascade (R is the reactant, I is the intermediate, and P is the product) including the loss pathways in red. (B) Diagram of a compartmentalized cascade with fluxes of reactant, intermediate, and product through the compartment barrier. (*F* refers to the flux of each component, and *r* refers to the rate of each enzyme.) (C) The dependence of the rates of various steps on the flux (i.e., diffusive conductance). (Adapted with permission from ref (197). Copyright 2019 American Chemical Society.)

Conceptually, the outflow of intermediates, I, from E1 can be considered to occur by two pathways: they are either channelled directly to E2 or lost to the bulk (see Figure 10A). The rate of catalysis by E2 depends on I arriving directly from E1 (channelled), or from the bulk. Therefore, the effect of channelling is apparent only when the concentration of intermediates in the surrounding volume is low. This situation applies at the beginning of the reaction (pre-steady-state), when there is a competing pathway that consumes the intermediate, or when the intermediate degrades.

The prediction stemming from this analysis, i.e., that proximity alone does not guarantee a rate enhancement, appears to contradict literature reporting that scaffolding enhances cascade rates compared to results obtained for free enzymes. Possible explanations for this apparent discrepancy are discussed below.

##### Clustering Increases the Chance That an Intermediate Reaches Another E2 before Being Lost to Bulk

Instead of an isolated pair of enzymes on a scaffold, there could be multiple molecules of E2 in close vicinity. In this case, the intermediates could be intercepted by another E2 (not necessarily the nearest E2) on the scaffold before being lost to the bulk. The effect of “clustering” (which was introduced in Section 3.3.2) was investigated by numerical simulation, treating the enzyme concentration as a continuous variable,^198−200^ or by a coarse-grained simulation, with each enzyme represented as a sphere with a patch to denote the active site.^201^ Intuitively, these simulations showed that a cluster of E2 molecules near E1 can enhance the cascade rate (up to a certain point when initial substrate supply becomes limiting), and the effect of clustering is greater when the intermediate is unstable. Hinzpeter et al.^202^ also investigated the effect of clustering, as well as the spatial organization within a cluster, and showed that clustering could increase the overall rate, but the effect is lessened in the regime where the enzyme rates are fast compared to diffusion, since substrate access to inner enzymes of the cluster becomes limiting.

##### Enhanced Channelling Due to Interaction between the Intermediate and the Scaffold

The analysis outlined in the previous section assumed no interaction between the intermediate I and the scaffold, but the proportion of intermediate entering the “channelled” pathway could also be enhanced by other factors.^203,204^ For reactions of horseradish peroxidase attached to a DNA scaffold,^205^ it was noted that the relative degree of rate enhancement (scaffolded vs free) depended on the organic reactant used by the peroxidase. The authors calculated the binding energy of each reactant with the DNA scaffold and concluded that an intermediate binding energy was beneficial due to increased local substrate concentration near the scaffold, but very strong binding might hinder access of the substrate to the enzyme itself. This mechanism has been invoked for a full cascade on a DNA scaffold,^172^ although the authors have stressed that catalytic enhancement could also arise instead from increased enzyme stabilization. Experiments^206^ and simulations^207^ on an artificial metabolon composed of hexokinase and glucose 6-phosphate dehydrogenase linked by an oligopeptide with glucose-6-phosphate as the intermediate, showed a decreased lag period (the time needed to reach a steady-state flux of intermediate), which is taken as an indication of a channelling effect. The lag period increased with increasing ionic strength of the electrolyte, consistent with enhanced channelling due to binding with a charged linker.

##### Changes to Microenvironment

The act of immobilization could lead to enhanced activities of individual enzymes within a cascade,^208−210^ thus increasing the overall cascade rate without any effect from channelling. Therefore, an important control experiment is to immobilize E1 and E2 on separate scaffolds, to confirm that the act of immobilization has not increased their rates.^211^ One of the key parameters which can affect enzyme rates is the local pH in the immediate vicinity of the scaffold, which can potentially deviate from the bulk pH under operating conditions.^212^ A proton-coupled reduction leads to consumption of protons, thus generating excess hydroxide ions at the electrode surface, with the degree of pH deviation from that of the bulk solution depending on the electrolyte buffering capacity, mass transport, and current density. The effect of local pH change near a DNA scaffold is not universal, and this has been investigated by separately immobilizing two enzymes with different optimal operating pH onto a DNA scaffold,^213^ in which the rate enhancement compared to free enzymes was similar in both cases.

#### 4.2.2 Kinetics of “Closed” Compartmentalized Cascades

In contrast to the previous section, in which the intermediates were assumed to diffuse freely in a single large volume, *compartmentalized* cascades comprise multiple copies of each enzyme E1 and E2 (see also the previous section on clustering) enclosed within a semipermeable wall that restricts the release of intermediates and products, albeit also retarding the entry of reactants.

Tsitkov and Hess developed a macro-scale model of a simple compartment-confined enzyme cascade^197^ based on a linear two-enzyme system: their report is very helpful as it combines intuitive reasoning with less-intuitive predictions and compares the results with approaches made by others. They consider the compartment (Figure 10B) to have pores that filter the entry of reactant and release of both intermediate and final product, their entry/exit rates being defined by a “diffusive conductance” which for a given pore size depends on the number present. The performance of the cascade is thus determined by how fast the product is released (assuming it can be detected immediately) compared to the rate that the intermediate (assumed to be unstable in the bulk medium, so it cannot return) is released. The most important factors are the number of enzymes E1 and E2 encapsulated (not their concentrations—diffusion between E1 and E2 being fast) and their individual activity parameters, assuming Michaelis–Menten kinetics. For a given set of enzyme numbers and activities, the rates of release of product and intermediate vary with diffusive conductance according to Figure 10C. At low permeability, the substrate consumption rate matches that of product release (the intermediate always being trapped for a sufficiently long time to undergo reaction with E2) whereas, at high permeability, the intermediate is released before it can be processed further. The rate of product formation thus passes through a maximum value.

Other models have been proposed, for example, one by Liu and co-workers^214,215^ that addresses the flux of small organometallic catalyst molecules in and out of compartments; however, these models are less applicable to the case of immobilized enzyme cascades. Hinzpeter et al. also used modeling methods to predict optimal compartment sizes and composition of the cascades within the compartment.^216^

The flux of reactants is a key parameter in compartmentalized cascades, and Adamson et al. demonstrated the ability to control the reactant flux by tuning the pore size of protein cages.^217^ They were able to vary the pore size as well as pore charge, and their molecular dynamics simulations indicated, as expected, that negatively charged pores would impede the flux of anionic reactants.

### 4.3 Energizing Enzyme Cascades

Bespoke artificial (cell-free) enzyme cascades have become an established area of biocatalysis with huge potential for synthesis of pharmaceuticals and intermediates.^218−222^ Reaction rates and performances are usually monitored by measuring the release of products or intermediates, since the scaffold is suspended in solution. Where required, the redox energy needed to drive a reaction is provided by a chemical such as formate or glucose, or light, as in the work of Erb and co-workers.^187^ By immobilizing a cascade at an electrode, it becomes possible to use electrochemical methods to study rates directly and gain control over the driving force and even the direction of reaction. Extending our earlier discussion, a DNA-scaffolded cascade was immobilized onto a Au electrode surface, allowing electrochemical detection and monitoring of the product generated by the cascade.^223^

Redox polymer films represent a configuration that combines mediated electron transfer with immobilized enzymes.^224−228^ The redox-active films contain covalently bound functionalities, such as viologens, the reduction potentials of which are close in value to their freely diffusing counterparts. For a redox polymer to mediate a reaction bidirectionally, its reduction potential must be close to that of the target enzymatic reaction. For example, for bidirectional 2H^+^/H_2_ interconversion in the pH range 6.4–8.8, an alkylated bipyridine-based polymer with a reduction potential of −0.43 V vs SHE was used.^227^ This concept was extended to drive a cascade by immobilizing ferredoxin-NADP^+^ reductase (FNR), as the NADPH regeneration catalyst, and a NADPH-dependent crotonyl-CoA carboxylase/reductase onto the polymer.^228^ Enzyme cascades are increasingly being driven electrochemically, even if they are not presented in a nanoconfined manner. Minteer and co-workers recently described an elegant enzyme cascade for activating inert hydrocarbons using electrochemistry mediated by neutral red dye.^229^ Their work demonstrated that the power of enzyme cascade biocatalysis is readily transferred into mainstream electrocatalysis.

## 5 The Electrochemical Leaf

### 5.1 Overview of the Discovery

The “electrochemical leaf” (e-Leaf) is a way of driving and channelling catalysis by an enzyme cascade loaded within a robust and scalable nanoporous electrode material. Crucially, nanoconfinement is combined with direct electrocatalytic NADP(H) interconversion by one of the trapped enzymes: the resulting NADP(H) recycling is fast, reversible, and localized for high efficiency. The e-Leaf enables an operator to energize, control, and observe cascade catalysis literally at the “touch of a button”.

**Figure 11:**
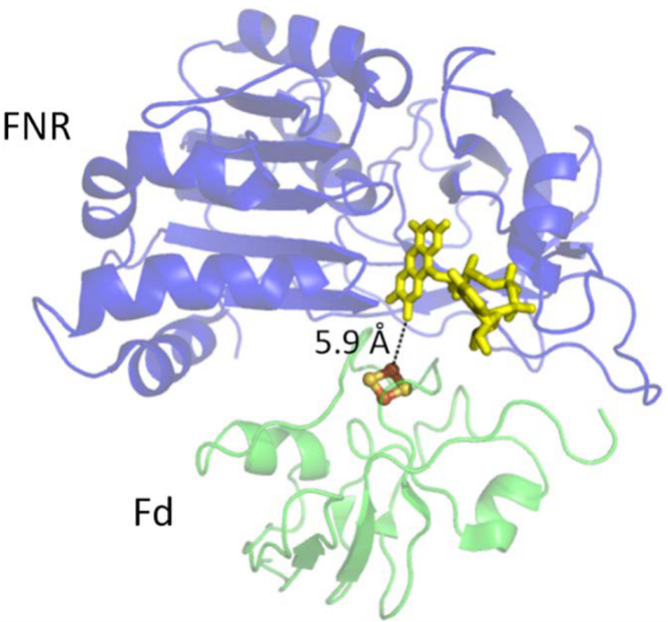
Structure of ferredoxin-NADP^+^ reductase (FNR) shown complexed with its natural electron donor, a ferredoxin (Fd) containing a [2Fe-2S] cluster. The minimal electron tunnelling distance between the [2Fe-2S] cluster and FAD serves as a guide as to how rapid electron exchange occurs when FNR is on an ITO surface (PDB: 1GAQ).

Two major enzymes are important for catalyzing the interconversion between electrons and NAD(P)(H) in biology. In catabolic pathways, NADH oxidation by membrane-bound quinones is catalyzed by NADH-quinone oxidoreductase (NQO): the mitochondrial enzyme is known as Complex I, and the energy released is used to pump protons across the inner membrane, which ultimately results in the production of ATP by ATP synthase. This giant enzyme consists of over 40 subunits, 14 of which are highly conserved and house FeS clusters (8 in total) that relay electrons from the flavin (FMN) active site (at which NADH is oxidized) to the site of quinone binding.^230^ A truncated form of NQO composed of the membrane-extrinsic domain exhibits reversible electrocatalysis of NAD^+^/NADH on a PGE electrode (Figure 5B), although the response is unstable.^29^ In photosynthesis, NADPH production in chloroplasts and green algae is catalyzed by ferredoxin NADP^+^ reductase (FNR) which was mentioned earlier.^231^ In contrast to NQO, FNR is a small, hydrophilic enzyme (39 kDa) having a single subunit and containing only a noncovalently bound FAD group: it receives electrons from Photosystem I via the small one-electron carrier protein ferredoxin (Fd), and its main role is to recycle NADP^+^ into NADPH for use by the Calvin cycle in which CO_2_ is assimilated into organic compounds. The structure of the FNR-Fd complex is shown in Figure 11.^232^

Ferredoxin-NADP^+^ reductase displays a reversible nonturnover signal when adsorbed on an indium tin oxide (ITO) electrode formed by depositing ITO nanoparticles on a support.^233^ Striking early observations were that the signal was located at the expected potential and had half-height widths of <60 mV for both reduction and oxidation directions, and the amplitude corresponded to multiple monolayers of enzyme. Upon introduction of NADP^+^, the nonturnover signal of FNR was amplified to produce peak-type cyclic voltammetry of the NADP^+^/NADPH couple.^233^

#### 5.1.1 Electrochemistry of FNR

**Figure 12:**
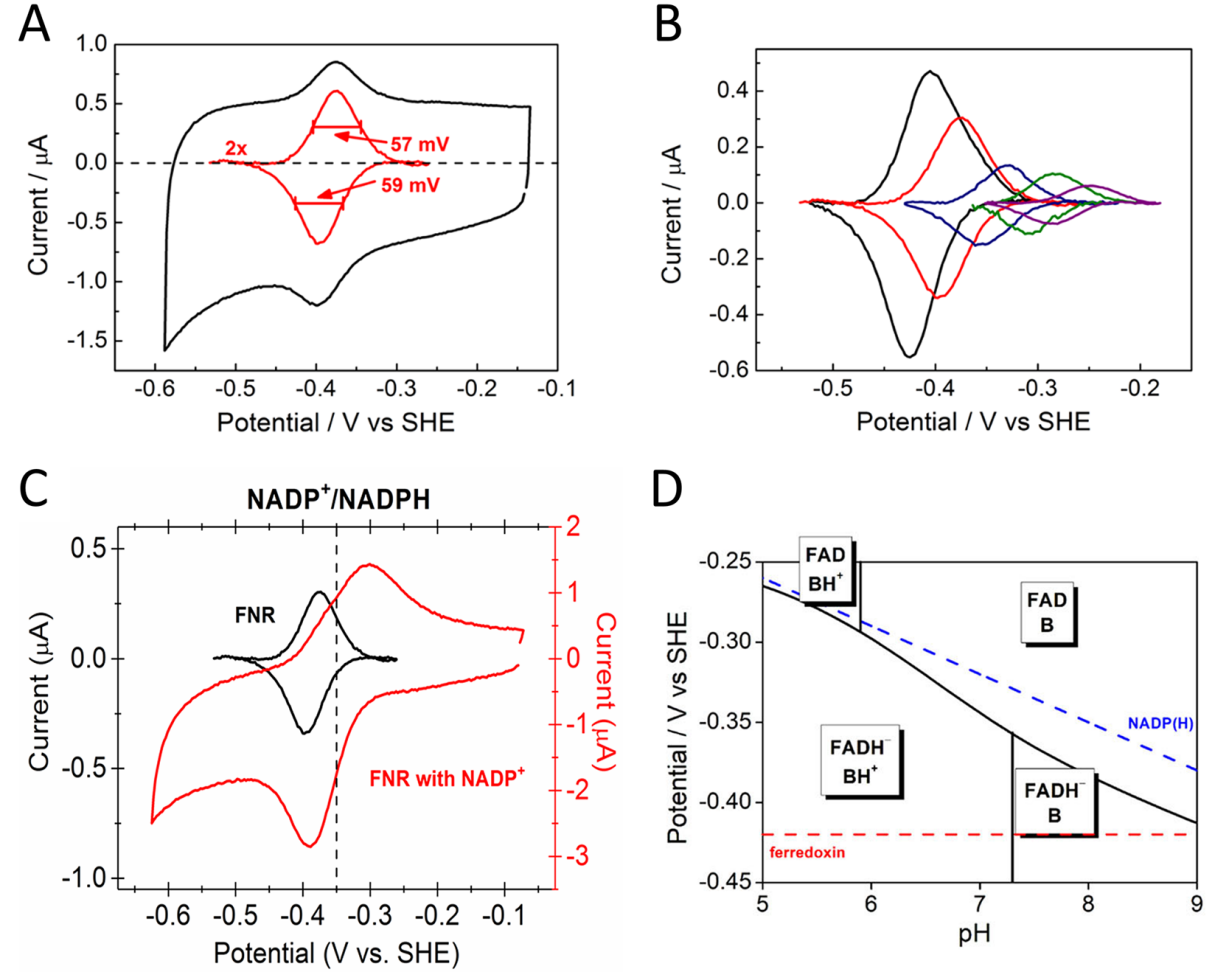
Electrochemistry of FNR and NADP^**+**^. (A) Cyclic voltammogram of FNR adsorbed in the pores of an ITO electrode (pH = 8), with background-subtracted peaks enlarged in current scale. (B) Background-subtracted cyclic voltammograms recorded at various pH values, 9, 8, 7, 6, and 5 (left to right). (C) Cyclic voltammogram recorded after addition of NADP^+^ to the solution (pH 8); the corresponding baseline-subtracted signal due to FNR (before adding NADP^+^) is also shown. (D) Pourbaix diagram for FNR with the pH dependence of the NADP^+^/NADPH couple included as a reference. The pH dependence of average peak potentials for FNR was obtained by fitting to a model that includes weak coupling to the protonation of a nearby base B. (Adapted with permission from ref (233) and reproduced with permission from ref (234). Copyright 2020 AIP Publishing.)

It was the detailed examination of this electrochemistry that led to the development of the e-Leaf, so named because the electrochemical recycling of NADP^+^/NADPH localized in electrode nanopores resembles the NADPH regeneration process occurring in chloroplasts. Important information was obtained by measuring how the nonturnover signal due to FNR varies as a function of pH. The FNR was allowed to adsorb from dilute solution onto an ITO electrode formed by electrophoretic deposition (see later), and several more detailed observations were made using cyclic voltammetry. The principal results are summarized in Figure 12: (i) The size of the signals increased as the pH was raised. (ii) The peaks broadened as the pH was lowered but remained symmetrical with half-height widths <90 mV. (iii) From the variation of average peak potential with pH, a Pourbaix diagram was constructed (Figure 12D).^7,234^

The results led to the following conclusions. First, the FNR coverages under all conditions were much higher than that possible for a monolayer, data at pH 8 suggesting that >100 monolayer equivalents were present with the value increasing further as the pH was raised to 9. The fact that the peaks remained sharp despite the high coverage meant that the FNR molecules cannot be laid upon each other, which would result in slow electron transfer, but must be buried deeply within the porous layer. Moreover, the FNR molecules must experience similar potential environments, otherwise the peaks would show a marked broadening due to inhomogeneity (dispersion). Second, the intimate association of FNR with the ITO surface inside the pores must somehow mimic its natural interaction with ferredoxin (Figure 11). Third, the width of the FAD redox signal, which approaches but does not attain the limit expected for two-electron cooperativity, could be explained in terms of a small but significant presence of the one-electron intermediate, as originally detected by spectroscopy, thus equipping FNR perfectly for its natural (and new) role in transducing two one-electron transfers and a single two-electron NADP^+^/NADPH interconversion. The pH dependence of the two-electron reduction potential could be fitted to a dependence involving FAD (2e^–^/1H^+^) and interaction with a nearby base B: comparison with the NADP^+^/NADPH potential showed the degree to which FNR is biased in favor of NADP^+^ reduction vs NADPH oxidation over the pH range 5–9. The electroactive coverage of FNR is highest at pH 9, at around 500 pmol/cm^2^: the amplitude of the voltammetric peaks decreases upon acidification to pH 7 but recovers upon re-alkalination, thus showing that the enzyme has been retained in the pores but with a weakening (at pH 7) of the intimate contact with the ITO that is needed for electron tunnelling. The FNR molecule must be dynamically bound in order to function, as the binding and release of one NADP(H) for every two electrons transferred requires that the enzyme–ITO contact is relaxed.

#### 5.1.2 Bidirectional NADP(H) Recycling by FNR

The transformation to electrochemical NADP^+^/NADPH interconversion is easily visible upon introducing small quantities of NADP^+^ to the cell solution, the CV with its peak-type waveform indicating a quasireversible electrode reaction. The sharper shape of the reduction peak relative to the oxidation peak probably reflects the catalytic bias that is provided by the more favorable energetics for the FADH → NADP^+^ direction (panels C and D).^7,234^ Closer inspection showed that NADP^+^ or NADPH is partially trapped in the electrode pores, as the scan-rate dependence indicated a surface excess. Most significantly, despite the FAD not being covalently bound and despite the transient disruptions to the structure of the small enzyme that must occur while NADP^+^ or NADPH bind and dissociate during each catalytic cycle, the electrochemistry was stable. The implication was that the FNR electrochemistry might be exploited in a new technology.

In the obvious next stage, it was demonstrated that the efficient NADP^+^ recycling could be used to drive a second enzyme reaction—the reductive amination of 2-oxoglutarate to give l-glutamate catalyzed by l-glutamate dehydrogenase (GLDH).^233^ Most significantly, it was further established that, in order to couple NADP^+^/NADPH interconversion to the second reaction, GLDH must also bind in the pores of the ITO electrode. This requirement was confirmed for several other dehydrogenases, all of which were taken up slowly from dilute solution, thus demonstrating that their binding at the electrode (and, likely, deep penetration into the pores) must be a spontaneous process. In subsequent work, the enzymes were often pre-mixed (in desired ratios and concentrated form) and co-loaded onto the electrode surface by painting and allowing a period of time to soak in (a simple procedure known as dropcasting). Electrocatalytic nanoconfinement was thus set to emerge as a new way to run enzyme cascades.^235^

### 5.2 Electrode—Preparation and Considerations

#### 5.2.1 Transport within Porous Electrodes

Porous electrodes have been well-studied for a large variety of applications such as batteries, fuel cells, and electrolyzers.^236,237^ The greatly increased surface area compared to a flat surface allows much higher catalyst loadings, although for reactants and products the tortuosity of the porous layer leads to smaller effective diffusion coefficients compared to bulk values.^238−240^ In the e-Leaf, other factors become important, notably the concentration of enzyme partners (as opposed to individual enzymes) and the entrapment of cofactors and intermediates (the latter being important in allowing an extended multienzyme cascade to operate efficiently).

#### 5.2.2 Construction and Characterization of a Porous ITO Electrode

As a commercially available transparent conducting oxide material, ITO is typically used as dense, thin films in optical applications. For the e-Leaf, however, a porous ITO electrode is normally made by electrophoretic deposition (EPD) of presynthesized ITO nanoparticles onto the supporting electrode (typically PGE, Ti foil, or ITO glass). In EPD,^241,242^ a voltage is applied between two electrodes in a suspension of charged particles; the particles migrate in the electric field and deposit onto one of the electrodes. In practice, ITO NPs are suspended in an acetone/I_2_ mixture which releases H^+^ ions that bind to the ITO surface, making them positively charged so they migrate to the cathode. A layer of ITO particles can be deposited onto large-area supports having complex geometry, provided they are sufficiently conductive. The EPD method is quick, simple, and reliable, the layer thickness increasing linearly with deposition time.^243^

Other methods are available to produce more elaborate structures in which the porous networks are better controlled, and this ability will be useful as the e-Leaf is developed further. Nanostructured ITO electrodes with different architectures can be made using polymer spheres as templates, either with preformed ITO particles or In and Sn precursors.^244−246^ Pillar-type ITO structures have been synthesized by 3D-printing,^247^ glancing angle deposition,^248^ or by templating using porous polycarbonate membranes.^249^

#### 5.2.3 Characterization and Pore Size Distribution

**Figure 13:**
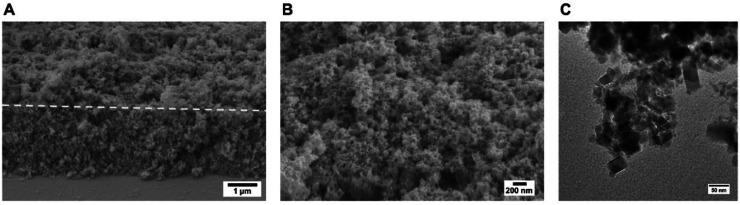
(A) Cross-section scanning electron microscopy (SEM) image showing a porous ITO electrode with ∼1 μm thickness. (B) Close-up SEM image. (C) Transmission electron microscopy (TEM) image showing individual ITO particles. The porous ITO layer was constructed by electrophoretic deposition of ITO nanoparticles onto ITO glass as a conductive support. The SEM was conducted on a Zeiss NVision 40 FIB-SEM instrument at an accelerating voltage of 5 kV, and the TEM was conducted on a JEOL 3000F instrument at an accelerating voltage of 300 kV.

The pore size distribution and porosity (i.e., void fraction) are important characteristics of a porous ITO electrode. Electron microscopy (Figure 13) shows that a layer produced by EPD (on an ITO glass substrate) contains a hierarchy of pore sizes with the largest on the scale of >100 nm.^250^ Enzyme molecules are thus able to enter the pores and may penetrate deeply and become trapped. Estimations of the void fraction created by electrophoretic deposition of identical spheres should be treated with caution, as real ITO particles, especially from commercial sources, have a significant particle size distribution (e.g., 10–50 nm diameter, as shown in Figure 13C).^235^ Nonetheless, it is instructive to know that numerical simulations of the EPD process have given a range of void fractions between 0.44 and 0.60, and the upper limit for the packing fraction of randomly packed identical spheres appears to be approximately 0.64 (corresponding to a lower limit of 0.36 for the void fraction).^251^

### 5.3 Operating Enzyme Cascades in ITO Nanopores

**Figure 14:**
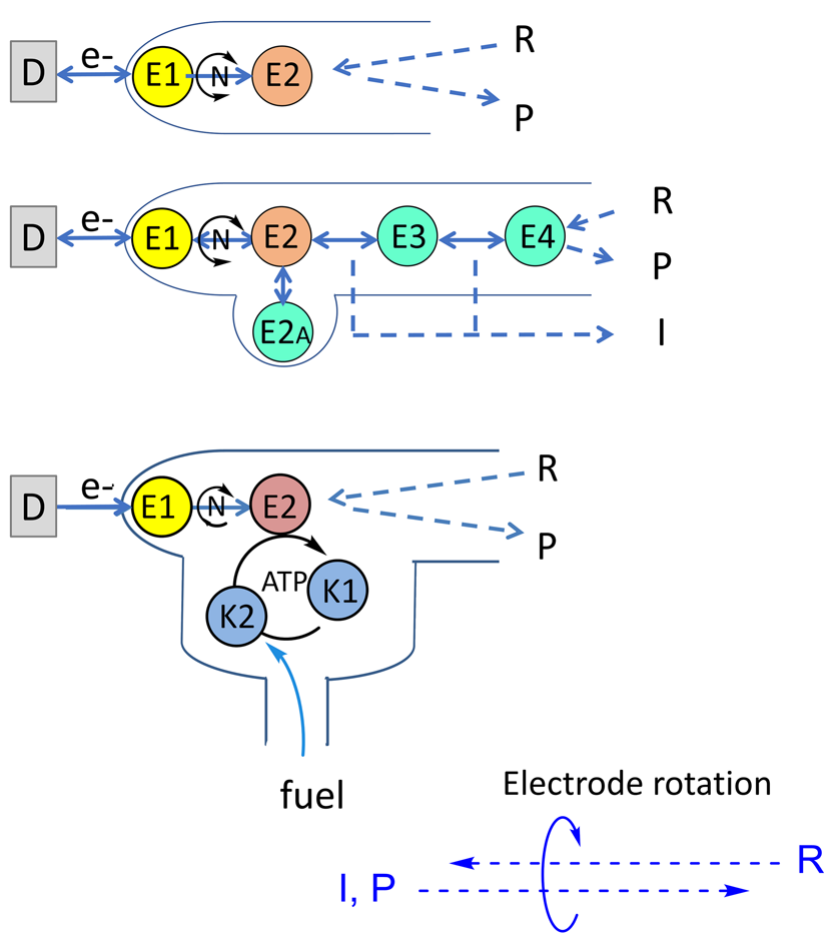
Examples of e-Leaf cascade maps. By analogy with urban transport maps, enzymes (E) are located according to their position in the cascade rather than their spatial arrangement in the electrode nanopore. E1 is the transducing enzyme (FNR); N = NAD(P)(H); E2 is a dehydrogenase; E3 and E4 are additional enzymes in an extended cascade. EnA are service enzymes such as carbonic anhydrase; K1 and K2 are kinases; and the fuel is a phosphorylating compound such as phosphoenolpyruvate (PEP). D stands for “dashboard”; it features the electrochemical workstation and accessories such as electrode rotator and reagent injection systems. Electrode rotation allows some control over the entry and exit of reactant (R), product (P), intermediate (I), and fuel.

A perceived shortcoming of the e-Leaf is that the scaffold is poorly defined; neither the spatial separation of the different enzymes nor their stoichiometry can be controlled or measured directly. As noted in section 4, the actual distances between the nanoconfined enzymes may have little importance in ensuring the efficient channelling of intermediates, as their diffusion on the nanoscale is expected to be much faster than enzyme catalysis.^158,159^ On the other hand, the porous ITO layer is a closed system: the number of enzymes entrapped per unit volume can be very high, and the ability to retard the release of exchangeable cofactors and intermediates should be highly advantageous. Although spatial relationships are obscure in the enzyme-loaded ITO layer, the sequential ordering of enzymes, E1, E2, ..., En, is known, and it is convenient to adopt an “urban transport map” approach to represent the electrochemically driven flow within e-Leaf cascades confined in an electrode nanopore. Enzymes (stations) are related by position, not distance: a product leaving one enzyme is automatically directed to the next enzyme along the line. Some basic cascades are shown in Figure 14, where “D” represents the dashboard—a combination of electrochemical workstation and manual inputs that is likened to the dashboard of a car. The electrode system is denoted (E1 + E2 + En)@ITO/support where E1 (as in section 4.2.1) is the first enzyme along the chain, but is now identified as FNR, and E2 is an NAD(P)(H)-dependent dehydrogenase; En ... represents further enzymes that may be included. The “support” is the underlying material, i.e., graphite, Ti foil, ITO glass, etc.

**Figure 15:**
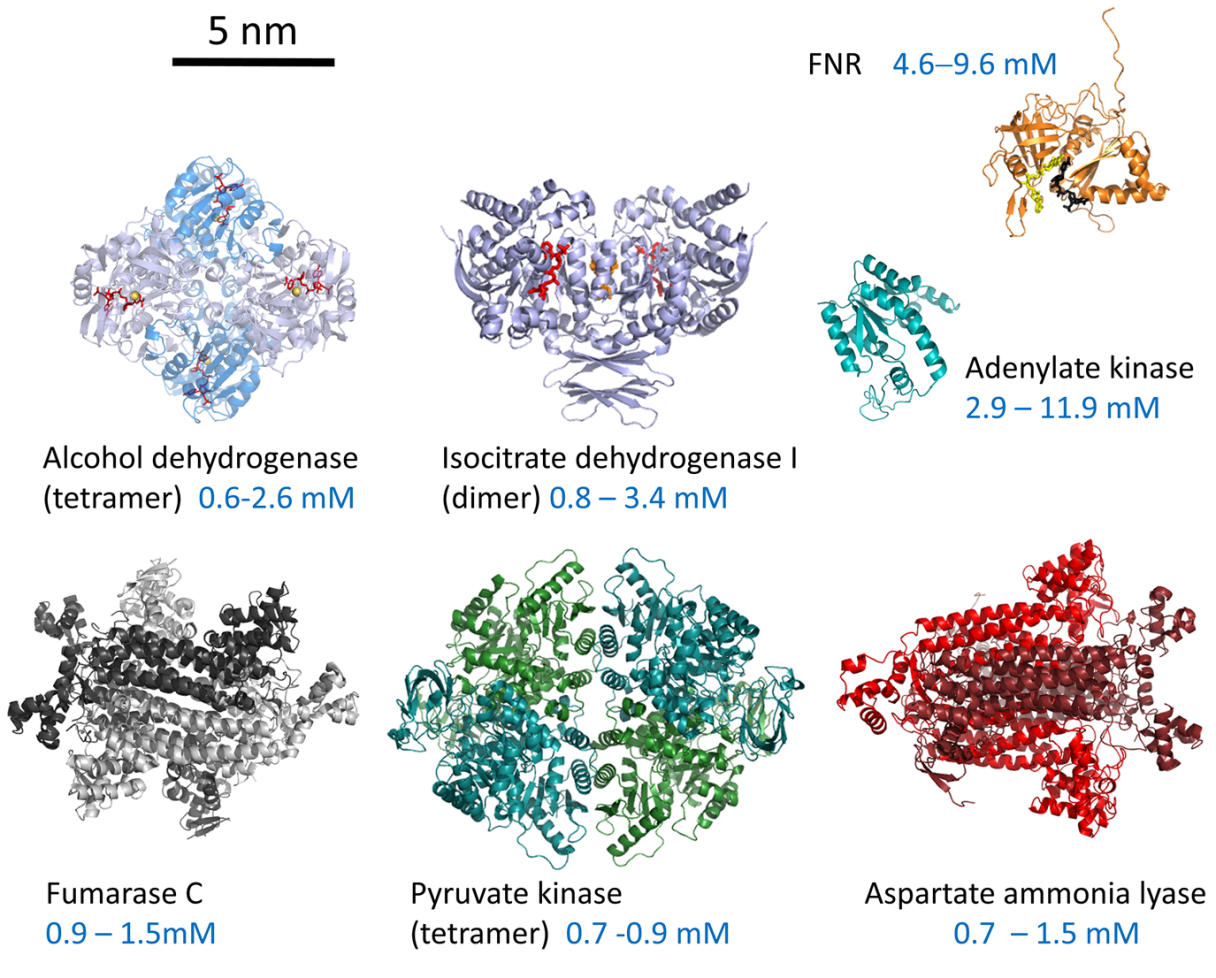
Structures and relative dimensions of some enzymes used in the e-Leaf. For each example, a maximum concentration range is given: the higher value is based on close packing of spheres, and the lower value is that expected for packing of crystallographic unit cells. PDB codes: FNR, 1QFZ; ADH, 7JNS; IDH1, 1T0L; AK, 2AK2; FumC, 3E04; PK, 1A49; AspAm lyase, 1JSW.

The thermodynamics governing the spontaneous concentration and entrapment of large enzyme molecules in electrode nanopores have yet to be investigated in detail. Even so, it is very instructive to consider reasonable concentration limits for packing enzymes into the pores of an ITO electrode. First, from section 5.2.3, if we assume a void fraction of 0.5 within a layer of 3 μm depth on a 1 cm^2^ electrode, the volume available to enzyme molecules that are able to penetrate the pores on that electrode is estimated at 3 × 10^–7^ dm^3^. Second, the maximum molar concentration possible for a specific enzyme can be estimated by assuming either that such a concentration is reached by close packing of equivalent spheres (based on average radius) or that it is achieved by stacking of crystallographic unit cells. The average center-to-center separation distance *d* (nanometers) between molecules in solution is obtained by a simple algorithm, *d* = 1.18/*C*^1/3^, where *C* is the molar concentration: thus, as a familiar reference, hemoglobin (average radius 5 nm) has an average center-to-center distance of 6.8 nm in red blood cells where its concentration is 5 mM.^253^Figure 15 compares the sizes of a selection of enzymes studied to date along with the maximum concentration range achievable in each case. The lower values for estimations based on unit-cell dimensions take greater account of irregular geometries and solvent.^254^ The average center-to-center distance of molecules at 1 mM concentration is 11.8 nm.

Taking the results outlined in section 5.1, a surface coverage for FNR of 100 pmol/cm^2^ would thus correspond to a concentration of 0.67 mM in a 3 μm layer; at 500 pmol/cm^2^, as recorded at pH 9, this value rises to 3.4 mM: the maximum concentration of FNR based on spherical close packing or unit-cell contact lies in the range 4.6–9.6 mM. We are thus confident that enzymes can become highly concentrated and crowded in the ITO cavities.

### 5.4 Exploiting Bidirectionality

An obvious advantage over other methods for operating enzyme cascades is the combined ability to energize, control, and observe a reaction in either direction. Bidirectionality can be exploited wherever the thermodynamics of the cascade reaction are well matched to those of NADP^+^/NADPH interconversion, examples being the numerous carbonyl/alcohol or carbonyl/amine interconversions that are used during the synthesis or purification of enantiomeric products. Such applications are important targets for the e-Leaf, but first, we summarize how deracemization or inversion reactions are carried out in more conventional ways.

#### 5.4.1 Deracemization and Inversion

Conventional methods for obtaining a pure enantiomer from a racemic mixture involve several steps, in addition to purification of the final product. Enzymes offer high enantioselectivity and conversion rates,^255^ and there are two main approaches to biocatalytic deracemization. The first of these methods is a one-pot multistep reaction, for example, the oxidation of a racemic mixture of secondary alcohols using a nonselective enzyme or chemical, followed by reduction of the ketone by a highly enantioselective enzyme. This method has even been successful using a single variant alcohol dehydrogenase (ADH), where the stereoselectivities of both steps were controlled by varying the amounts of cosubstrates for the enzymatic reactions.^256^ The second approach uses simultaneous dynamic kinetic resolution (DKR), which exploits the different rates of reaction for each enantiomer which are in equilibrium with each other and requires a highly efficient kinetic resolution step combined with *in situ* racemization. As the faster reacting enantiomer is depleted in the kinetic resolution step, the equilibrium concentrations of the two enantiomers are constantly changing by racemization of the slow-reacting enantiomer; DKR need not involve enzymes for either step but more often does, either exclusively or in conjunction with chemical catalysts.^257,258^

In such conventional catalysis or enzymology, it is difficult to drive a reaction first in one direction and then in the other, i.e., general oxidation followed by selective reduction, by simply reversing a condition. Altering conditions to reverse the direction of catalysis involves replacement of reagents, often with removal of the chemicals needed to operate in the initial direction. Photocatalysis presents another problem: light-driven reactions are unidirectional, although a process might be driven in the opposite direction by switching the source from reducing (donor) to oxidizing (acceptor). In contrast, electrocatalysis can be bidirectional if the primary step is reversible and other steps are thermodynamically compatible. Two examples have been investigated.

#### 5.4.2 Deracemization and Inversion by the e-Leaf

**Figure 16:**
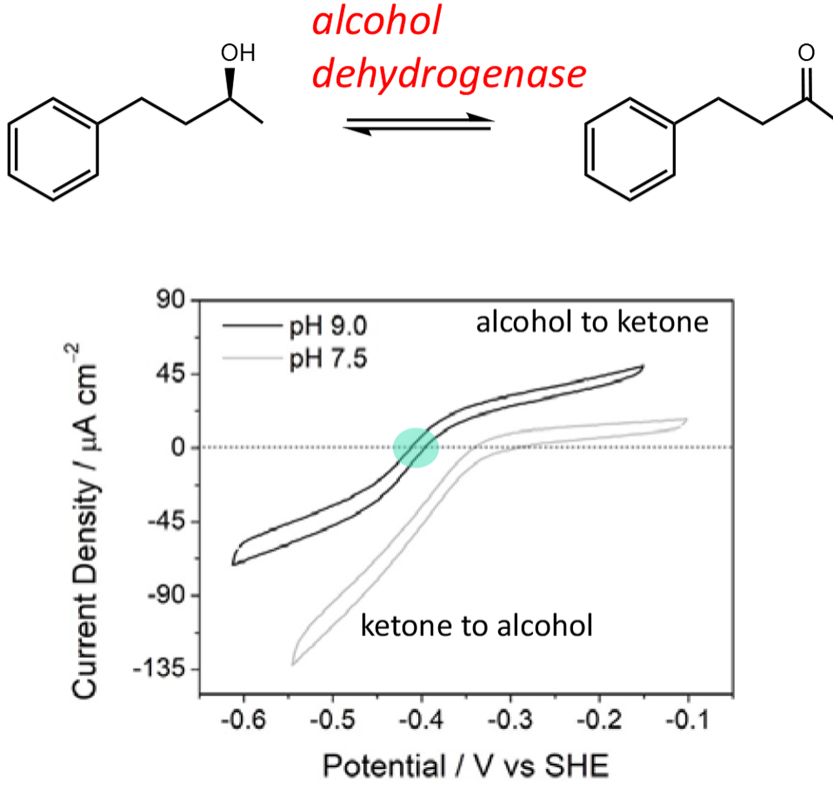
Bidirectional interconversion between secondary alcohol and ketone catalyzed by an alcohol dehydrogenase. The voltammograms shown in the lower panel are recording the electrocatalytic interconversion of a 50/50 mixture of 4-phenyl-2-butanone and (*rac*)-4-phenyl-2-butanol, occurring at a (FNR+ADH)@ITO/graphite rotating electrode. At pH 7.5, there is a strong catalytic bias in favor of reduction, but at pH 9.0, the currents in each direction become similar (allowing for some suppression of the oxidation by ketone, a product inhibitor). The formal potential of the ketone/alcohol redox couple (indicated by a green circle) is well-defined at pH 9.0. (Adapted with permission from ref (260). Copyright 2021 American Chemical Society.)

The e-Leaf offers a new and potentially simple approach to deracemization and inversion. Bidirectionality, coupled with enzyme nanoconfinement and the ability to observe the process in real time (thus informing the user about if and when to intervene), has been exploited to drive and control enantioselective interconversions between a ketone and secondary alcohol enantiomers, catalyzed by alcohol dehydrogenase (ADH) variants. Initial experiments involved the use of a single electrode loaded with FNR and variants of an ADH from *Thermoanaerobacter ethanolicus* (*Te*) having high (but not total) selectivity for the *S*-alcohol.^259^ At pH 7.5, the catalytic interconversion between 4-phenyl-2-butanol and 4-phenyl-2-butanone carried out using a (FNR + *Te*W110S)@ITO/PGE rotating disc electrode is strongly biased in favor of the reduction direction (Figure 16). Following the principles summarized in section 2, this bias arises for two reasons: first, the ketone is a product inhibitor during oxidation, and second, the NADP^+^/NADPH formal potential lies negative of the ketone/alcohol potential. Raising the pH to 9.0 corrects the latter factor, and a cyclic voltammogram obtained in the presence of equal concentrations of ketone and alcohol appears both bidirectional and reversible, noting the sharp cut through the zero-current axis made at −0.41 V.

**Figure 17:**
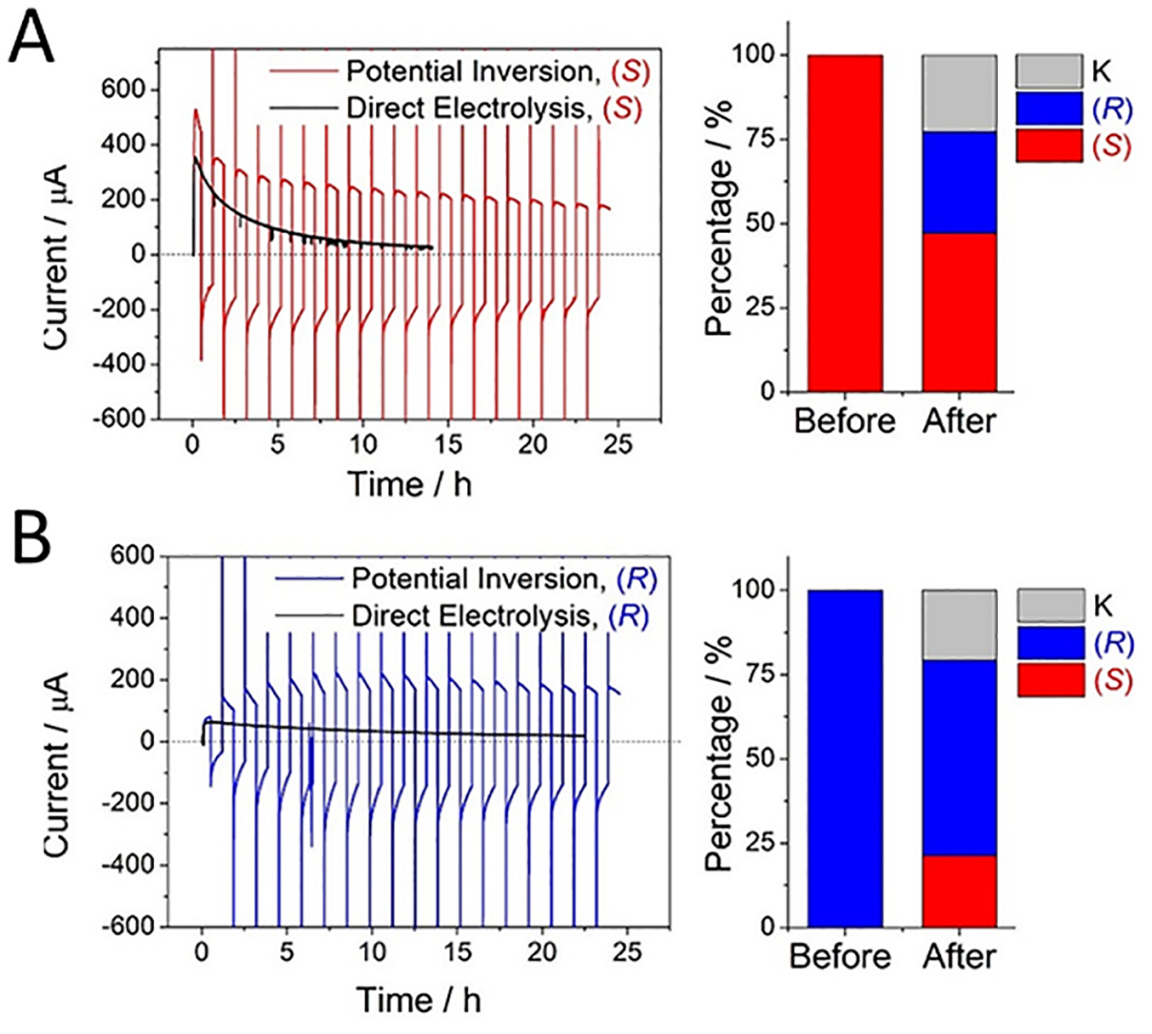
Nanoconfined electrocatalysis promotes racemization. Effect of periodic potential inversions (reduction at −0.65 V, oxidation at −0.11 V vs SHE, pH 9.0) on the product distribution generated by interconversion between 4-phenyl-2-butanol and 4-phenyl-2-butanone, using a (FNR+TeW110A)@ITO/Ti foil electrode, starting from (A) (*S*)-4-phenyl-2-butanol or (B) (*R*)-4-phenyl-2-butanol. In each case, the potential oscillations accelerate racemization (the thermodynamically favored outcome). TeW110A has high but not total selectivity for the *S*-enantiomer; accordingly, oxidation and reduction currents for the R-enantiomer each increase during the first 5 cycles (7 h) whereas, for the S-enantiomer, there is a marked decrease in oxidation current over the first five cycles, while the reduction current increases. Eventually, after 15 h, the oxidation and reduction currents level out and become equal for A and B. (Adapted with permission from ref (260). Copyright 2021 American Chemical Society.)

Experiments were carried out to test the effect of periodically switching the electrode potential (vs SHE) between −0.11 V (oxidizing) and −0.65 V (reducing) using a solution of either the (*R*)- or the (*S*)-form of the alcohol at pH 9.0 (Figure 17); importantly, regardless of the starting reactant, the product was a racemic mixture—the thermodynamically favored outcome (Δ*G* = −*RT* ln 2, i.e., −1.7 kJ mol^–1^ at 298 K). Whereas the intention had been to convert both enantiomers into ketone during the oxidative steps but produce mainly the *S-*alcohol during the reductive steps, it appeared that within the nanoporous environment the enzyme-catalyzed reactions are so fast and reversible as to favor equilibration during the course of each period.

A deracemizer was constructed by using two ADH variants with opposing enantioselectivities, each one being loaded into a separate electrode. Racemic 4-phenyl-2-butanol was first oxidized at −0.11 V using a (FNR + *Te*W110A)@ITO/Ti electrode at pH 9.0; the pH was then adjusted to 7.5, the electrode replaced by a (FNR + ADH LK)@ITO/Ti electrode (ADH LK being the (*R*)-selective enzyme from *Lactobacillus kefir*) and reduction carried out at −0.65 V. This sequence resulted in the production of (*R*)-4-phenyl-2-butanol at 92% enantiomeric excess (ee). By switching the sequence, i.e., oxidizing with (FNR + LDH LK)@ITO/Ti and reducing with (FNR + *Te*W110A)@ITO/Ti, (*S*)-4-phenyl-2-butanol was produced at 99% ee.

**Figure 18:**
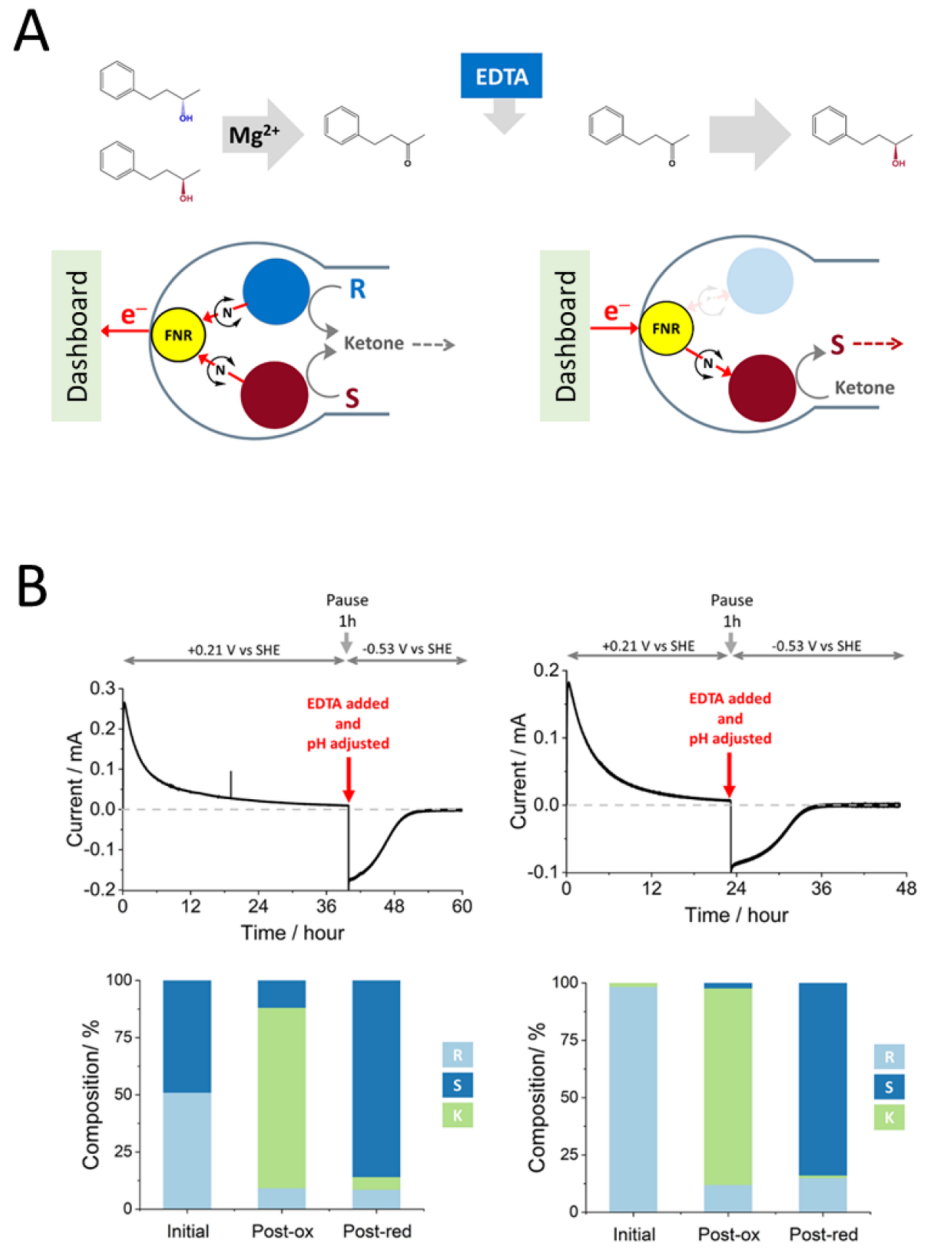
Deracemization and inversion in the e-Leaf. (A) Scheme showing the control of two alcohol dehydrogenase enzymes with opposing enantioselectivities, nanoconfined and driven in the porous electrode of the e-Leaf. The system has dual control: electrochemical control allows the system to be driven bidirectionally, and control by selective metal ion activation allows the R-specific enzyme to be switched off by the addition of EDTA to chelate Mg^2+^ ions, which it requires in order to function. (B) Chronoamperograms showing deracemization (left) and inversion (right). In both experiments, an oxidizing potential is first applied, causing both enzymes to convert their corresponding alcohol enantiomer to ketone at which point the R-specific enzyme is switched off by the chelation of Mg^2+^. A reducing potential is then applied to drive the S-specific enzyme to reduce the ketone produced during the first phase. Bar charts show the composition at each stage. (Adapted with permission from ref (264). Copyright 2022 Royal Society of Chemistry.)

The ADH from *Lactobacillus kefir* is not a Zn enzyme—the mechanism involving instead a Ser–Tyr–Lys catalytic triad; however, its activity is strictly dependent on the binding of two noncatalytic Mg^2+^ ions in the tetrameric structure.^261−263^ This distinction with the more common ADHs that depend upon a tightly bound catalytic Zn^2+^ and do not contain Mg^2+^ offered a way to create a deracemizer or inverter (*R* → *S*) using a *single* (FNR+ *Te*W110A+ADH LK)@ITO/Ti electrode. All that was required at the half-way stage (after the oxidation half-cycle) was to adjust the pH and introduce EDTA to remove Mg^2+^ from ADH LK, thus “silencing” it for the reduction direction (Figure 18).^264^

### 5.5 Extended Cascades

#### 5.5.1 Four-Enzyme Linear Cascade

**Figure 19:**
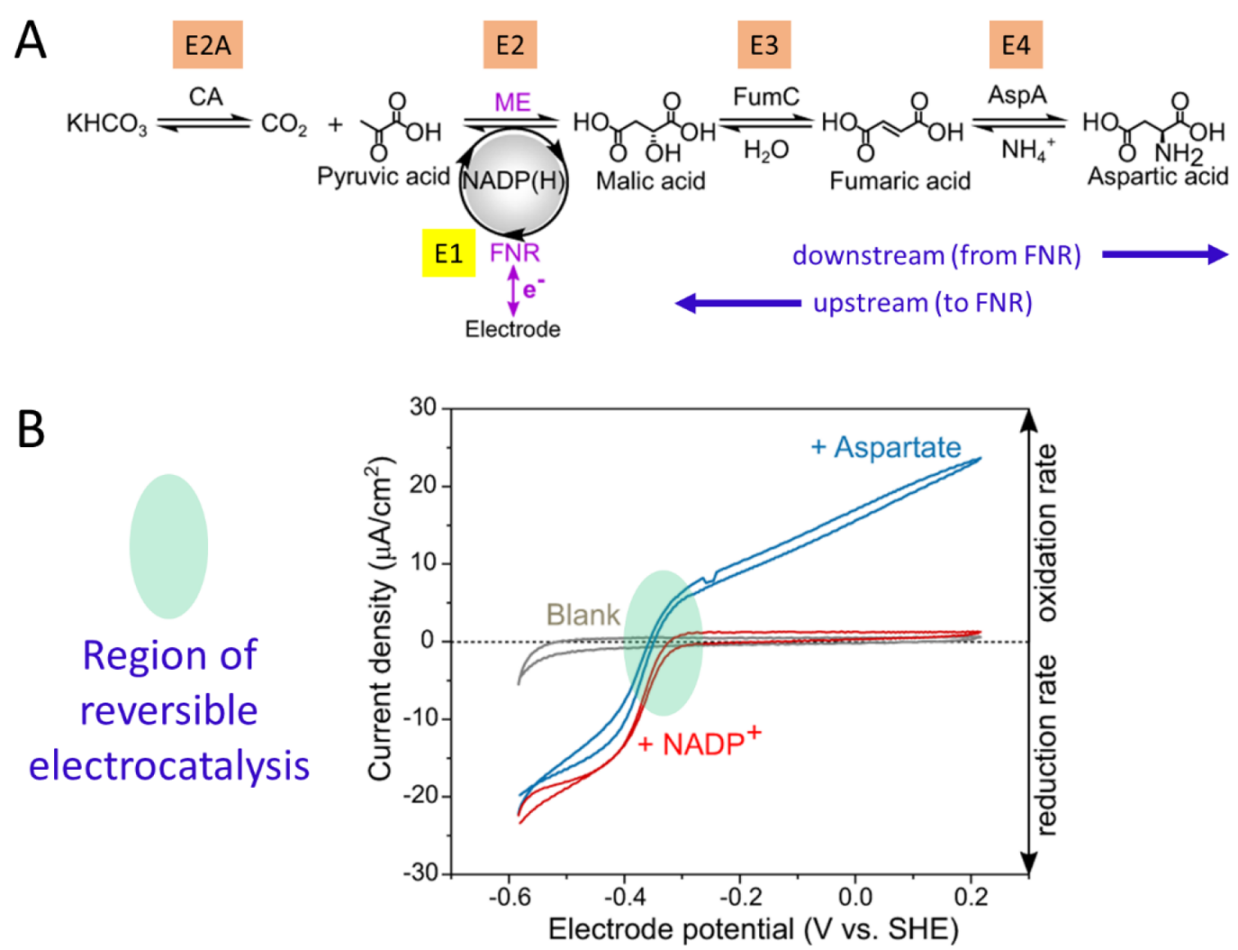
Cyclic voltammetry of an extended enzyme cascade trapped in electrode nanopores. (A) Nanoconfined cascade for the interconversion between pyruvate and aspartate. CA, carbonic anhydrase (E2A); FNR, ferredoxin-NADP^+^-reductase (E1); ME, malic enzyme (E2); FumC, fumarase (E3); AspA, aspartate-amino-lyase(E4). (B) Cyclic voltammetry (25 °C, pH 7.5, 1 mV s^–1^) of the 5-enzyme cascade (electrode loaded in ratio 0.1 CA/1 FNR/5 ME/1 FumC/1 AspA) in buffer 0.05 M HEPES, 20 mM pyruvate, 0.1 M KHCO_3_, 0.1 M NH_4_Cl, 4 mM MgCl_2_, 1 mM MnCl_2_. Gray: blank, no cofactor present. Red: after injection of NADP^+^ (to 20 μM). Blue: after injection of aspartate (to 20 mM). (Adapted with permission from ref (266). Copyright 2021 Springer Nature.)

An example of an extended linear cascade, featuring four “main-line” enzymes and one “service branch”, is represented in Figure 19. In the reductive direction, malate dehydrogenase (decarboxylating) (E2, “malic enzyme”) catalyzes the reaction of pyruvate with CO_2_ that is produced *in situ* from bicarbonate ions, through catalysis by carbonic anhydrase (E2A). The malate intermediate is dehydrated by fumarase (E3), and the fumarate intermediate is finally aminated by l-aspartate ammonia lyase (E4). For the oxidative direction, the order is simply reversed. The cascade is an extension of the two-enzyme system originally designed to demonstrate the reductive incorporation of CO_2_ into an organic compound using light or electricity.^265^ The extended cascade produced valuable insight into how nanoconfinement impedes the escape of intermediates and highlighted the different results that are obtained when the direction of the cascade is reversed.^266^

**Figure 20:**
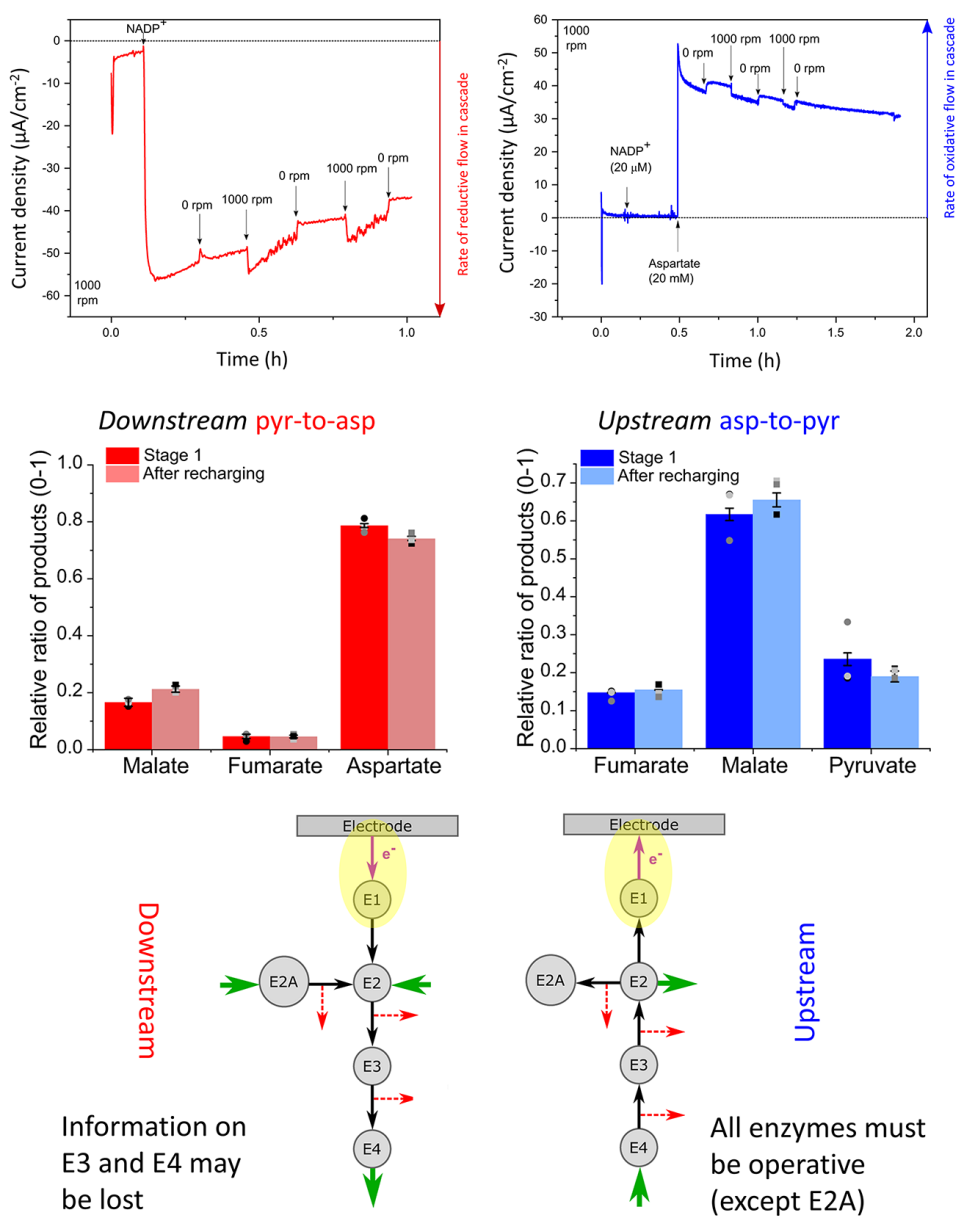
Extended cascades and the information that is available from the electrocatalytic rate: the bidirectional pyruvate/malate/fumarate/aspartate system, as an example. (A) Rotating the electrode increases the rate of catalysis of pyruvate reduction (left) but decreases the rate of catalysis of aspartate oxidation (right). (B) Relative percentage of products and intermediates released during (left) pyruvate reduction and (right) aspartate oxidation. The slowest step is malate oxidation. (C) The information obtained from the electrocatalytic current depends on direction (E2A is carbonic anhydrase catalyzing a CO_2_/HCO_3_^–^ “service” branch). During pyruvate reduction, further decisive steps occur *downstream* of E1/E2 coupling and may not influence the current. During aspartate oxidation, decisive steps occur *upstream* of E1/E2 coupling, and the current is strongly influenced. (Adapted with permission from ref (266). Copyright 2021 Springer Nature.)

The pyruvate–aspartate cascade was studied using a combination of electrochemical and NMR analytical methods. By using a rotating disc electrode to compare the influence of mass transport on electrocatalytic rate in each direction, it was clear that the oxidation and reduction reactions were affected differently: whereas the rate of reduction of pyruvate increased slightly when the rotation rate was stepped from 0 to 1000 rpm, the rate of oxidation of aspartate diminished slightly. These changes were reversed when the rotation rate was returned to 0 rpm. The results are shown in Figure 20 together with analyses by NMR of the various reactants, products, and intermediates released into solution as the process was driven in either direction.

The effects of electrode rotation can be understood in terms of its small influence on the diffusion layer gradient close to the ITO surface. Driven in the direction of pyruvate reduction, the current increases because rotation enhances the transport of the reactant, pyruvate, to the electrode; any enhancement of the rate of escape of intermediates is not detected because their processing occurs after the primary cofactor recycling step. In contrast, for aspartate oxidation, the decrease in current arises from the enhanced escape of intermediates that must be processed in order to produce the malate that is required at the final cofactor recycling step. The information given by the current thus depends on whether a step is *upstream* of the primary recycling step (and thus detected) or *downstream* (and not detected unless there is buildup of an inhibitory intermediate). The analysis results show that the reduction of pyruvate to aspartate, which involves assimilation of both CO_2_ and NH_4_^+^, is very efficient, whereas the oxidation of aspartate to pyruvate is affected by the release of a significant fraction of the malate intermediate before it can be oxidized, malate dehydrogenase being the least active enzyme along the cascade.

#### 5.5.2 Four-Enzyme System Combining NADPH and ATP Recycling

**Figure 21:**
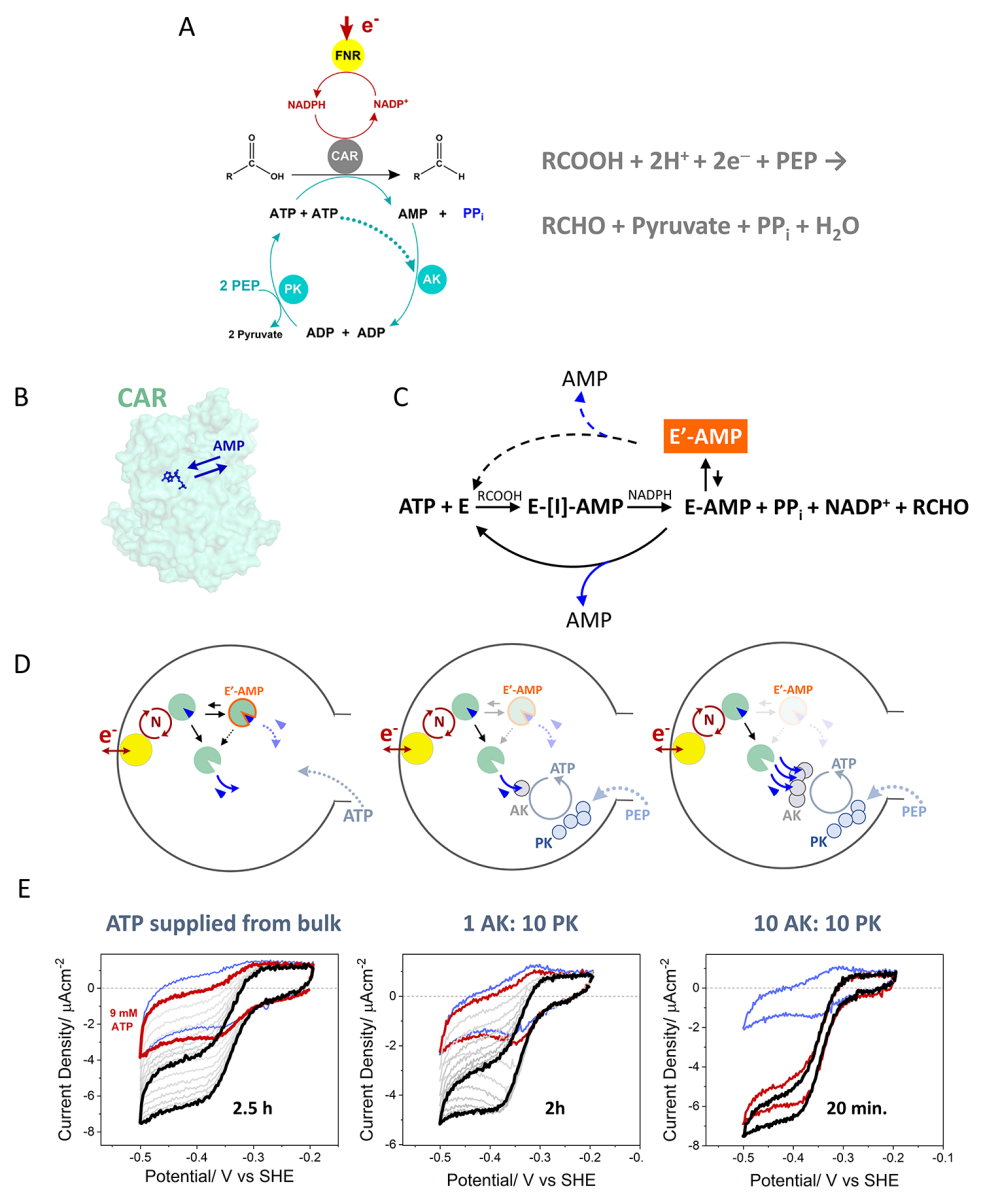
Confocal recycling of NADP(H) and ATP by a nanoconfined cascade in the e-Leaf. (A) Flowchart showing the cascade enzymes and reactions including FNR (which transduces electricity to regenerate NADP(H)), the kinase pair (adenylate kinase and pyruvate kinase) which transduces the chemical energy supplied as a fuel in the form of phosphenolpyruvate (PEP), and carboxylic acid reductase (CAR) which catalyzes the reduction of a carboxylic acid to an aldehyde when simultaneously energized from both sources; the overall reaction is written on the right. (B) Crystal structure of CAR with AMP tightly bound (PDB 5MSS). (C) Possible outcomes throughout the catalytic cycle of CAR as described in the main text. (D) Illustrations of the pore without the nanoconfined kinase cascade, which is therefore reliant on a supply of ATP from the bulk solution (left); with the kinase cascade present with a low level of AK (middle) and with a high level of AK (right). (E) Corresponding cyclic voltammetry experiments aligned with each pore: (left) ATP (∼9 mM) was supplied in the bulk solution; (middle and right) only AMP supplied, the system thus relying on the trace ATP contaminant. (Adapted with permission from ref (252). Copyright 2022 American Chemical Society.)

A dehydrogenase reaction requiring ATP as well as NADPH led to the construction of a nonlinear cascade in which the recycling of both cofactors is *confocal*; i.e., they occur in the same place. Carboxylic acid reductase (CAR: E.C. 1.2.1.30) catalyzes the reduction of carboxylic acids to their corresponding aldehydes in a reaction that requires both NADPH and ATP, the latter being converted to AMP. The reduction of cinnamic acid to cinnamaldehyde is one such reaction. A nonlinear cascade was designed, consisting of FNR, CAR, and a kinase pair to recycle the ATP (pyruvate kinase, PK, E.C. 2.7.1.40, and adenylate kinase, AK, E.C. 2.7.4.3). It was reasoned that such a cascade would be driven simultaneously by electrical energy transduced by FNR and chemical energy (supplied as a fuel in the form of phosphoenolpyruvate, PEP, contained in the cell solution) transduced by the kinase pair (Figure 21).^252^

Without the in-pore ATP recycling system, i.e., using a (FNR + CAR)@ITO/PGE electrode and introducing ATP as a stoichiometric reactant, it was noted that a very high concentration of ATP in solution (>5 mM) was required to produce a sizable catalytic current (Figure 21E, left). In stark contrast, when the kinase pair was coentrapped in the pores, i.e., using a (FNR + CAR + AK + PK))@ITO/PGE electrode, a similar final current density was achieved using only a trace amount of ATP [supplied initially at 10 μM in the bulk and even when omitting ATP completely and relying only on its presence as a trace contaminant in commercial AMP (Figure 21E, middle and right)]. The result proved that *in situ* recycling of ATP by the co-nanoconfined kinases gives a far superior catalytic system. The increase in current with successive scans was attributable to the accumulation (from the trace level of ATP in the commercial preparation of AMP) and recycling of ATP until a steady state was attained - the time-course depending on the amount and ratio of the coentrapped kinases (ranging from 20 min when both were loaded at high amounts to 3.7 h when both were loaded at very low amounts).^252^

The different shapes of the initial voltammograms obtained with and without the ATP recycling system provided additional insight. For the experiments featuring *in situ* recycling, the peaklike voltammograms obtained initially (and signifying depletion of a reactant) grew in magnitude upon successive scans, eventually becoming sigmoidal. The only component that can be depleted during the early scans is ATP, present initially at a level too low to supply both CAR and AK. The early scans thus represent a priming period, during which the action of the kinase pair develops with time (production of ATP by PK/PEP depends on the production of ADP by AK/AMP-ATP). Finally, the local ATP concentration reaches a level at which it is no longer depleted. This conclusion was further supported by separate chronoamperometric experiments in which a growth in current observed upon initiation by PEP no longer occurred with subsequent refuelings (since the kinase cycle is working maximally by that stage).^252^ In the experiment in which ATP was supplied as a bulk reactant and the kinases omitted, the voltammogram was sigmoidal from the start, changing only by an increase in current magnitude over time (Figure 21E, left). This unexpected gradual increase in current could not be due to diffusion of ATP from the bulk solution into the pores, since this had been established as a fast process. An explanation of the profound difference between introducing ATP as a reagent vs confocal NADPH/ATP recycling stems from the fact that CAR copurifies with a molecule of AMP bound,^267^ indicative of extremely tight binding that stabilizes a resting state of the enzyme (in steady-state kinetic studies, AMP is otherwise only a weak inhibitor).

A proposal for how *in situ* accumulation and recycling of ATP leads to a superior system, compared to that in which ATP is supplied as a bulk reagent, is illustrated in the pore sketches in Figure 21D. The scheme (Figure 21C) shows the possible outcomes during the catalytic cycle of CAR: after NADPH binds to the enzyme species with the carboxylic acid intermediate and AMP both bound, CAR catalyzes the reduction step, producing the aldehyde. At this point, the resulting E-AMP either releases AMP to regenerate active CAR or switches to a “resting inactive state” E′AMP (shown in orange) in which AMP is more tightly bound (the state revealed in the crystal structure, Figure 21B). The superior results obtained with the nanoconfined kinase pair, including removal of the lag period, result from the sequestration of AMP by AK and the subsequent accumulation and recycling of ATP by the kinase pair. A negative effect of nanoconfinement is to hinder the escape of AMP, allowing it to rebind to CAR; thus, in the absence of the kinase cascade, the probability that the inactive E′AMP state persists in the pores is high since AMP sequestration by AK is not possible. Use of a two-enzyme kinase cascade rather than a single enzyme system (e.g., a polyphosphate kinase) allowed for the deconvolution of the separate roles played by AMP sequestration and ATP accumulation and recycling as indicated in the experiments where AK is included either at a low level (1 AK:10 PK) or at an equally high level to PK (10 AK:10 PK), the latter reaching a maximum steady state much faster.

### 5.6 Scaling up and Widening the Scope

In order to scale up the e-Leaf for synthesis, it is important to compensate for the fact that heterogeneous catalysis occurs only at a surface, so the geometric (macroscale) surface area must be greatly increased; this requires an inexpensive and versatile electrode material and (ultimately) modular designs following the principles of batteries and electrolyzers. The e-Leaf has been successfully scaled up to build a 0.5 L batch reactor suited for small-scale synthesis. In a test run using l-glutamate dehydrogenase (GLDH) as E2, >99% of 20 mmol of 2-ketoglutarate (2.92 g) was converted to l-glutamic acid in 1.5 days. The electrode consisted of Ti foil sheets coated each side with the ITO layer housing FNR and GLDH, giving a total surface area of 525 cm^2^. With 10 μM NADP^+^ in solution, the total turnover number - TTN (the number of turnovers of NADP(H) per product molecule produced) was 3750.

**Figure 22:**
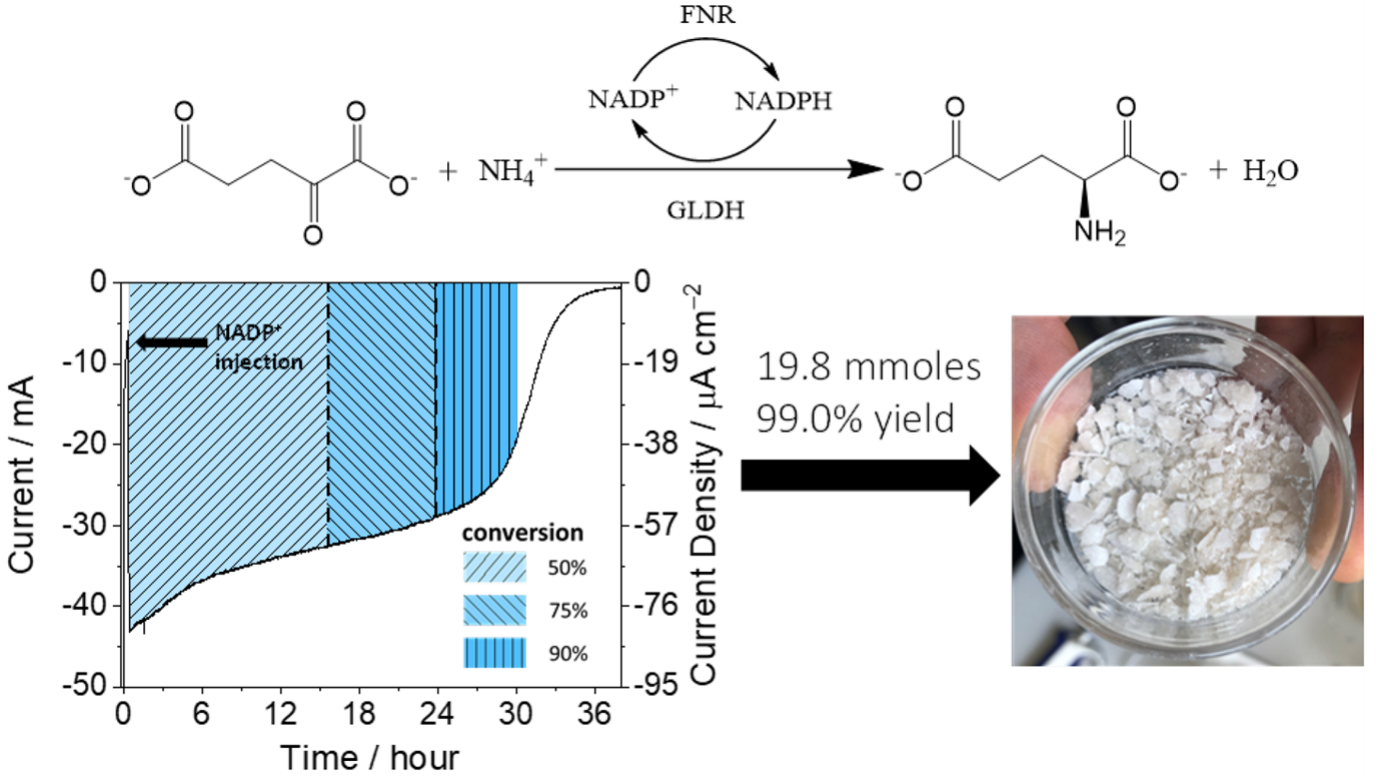
Feasibility of scaling toward pharmaceuticals. “Model” synthesis of l-glutamate from 2-oxoglutarate and NH_4_Cl using 10 double-sided (FNR+GLDH)@ITO/Ti foil electrodes, each of dimensions 7.5 × 3.5 cm, giving a total surface area of 525 cm^2^. The enzymes were loaded by drop-casting. The electrodes were housed in a stirred 0.5 L main reactor cell. Reactant concentrations: [2-oxoglutarate] = 40 mM, [NH_4_Cl] = 80 mM, adjusted to pH 7.5 with no added buffer, [NADP^+^] = 10 μM. Temperature, 25 °C; electrode potential held at −0.59 V vs SHE. The progress of the reaction is monitored continuously (left). A pure product can be obtained at the end of the reaction (right). (Adapted with permission from ref (268). Copyright 2020 Wiley.)

The whole process could be monitored continually, and the result is shown in Figure 22. The maximum current of 40 mA achieved early in the synthesis equates to a rate of 1.8 mg of product converted per minute. The current remains stable at a high level throughout the reaction and eventually drops sharply as the 2-oxoglutarate is depleted. The final product, l-glutamic acid, was extracted as crystals of high purity, there being few other chemicals present in the reactor solution.^268,269^

The e-Leaf thus offers a new way to synthesize high-value products using catalysis by enzyme cascades, all components of which can be loaded into an inexpensive ITO coating deposited easily on both sides of any number of plates of Ti foil serving as conductive supports. The two key engineering issues for scaling up are reaction optimization and reactor design. The abilities to control a reaction and monitor its progress continuously make it easy to evaluate conditions such as pH, enzyme loadings and ratios, temperature, potential, and concentrations of reactants and cofactors, all with a view to optimizing the conditions needed to achieve pilot scale and larger reactors. With regard to reactor design, the Ti foil electrodes (thickness 0.13 mm) can be scaled up into any shape or size and used as multiples. In general, costs of materials are low: the Ti foil is reusable, as the spent ITO coating is easily removed by mild acid treatment without damaging the support, and ITO nanoparticles (which may also be recycled) are produced commercially on a large scale.^268^ With cofactor TTNs set to exceed 10 000, the main expense may be in producing the enzymes, although these are likely to be overexpressed and only required in small amounts.

There has been great interest in improving cofactor regeneration systems, for both NAD^+^/NADH and NADP^+^/NADPH, with successful outcomes relying on formate/formate dehydrogenase for NAD(H) and glucose/glucose dehydrogenase for NADP(H). Each of these systems is simple and reliable but limited in terms of bidirectionality and interactive monitoring. By replacing the C-terminal tyrosine by a serine, the activity of FNR can be switched to NAD(H) instead of NADP(H).^270,271^ This modification (and probably others in turn) means that the e-Leaf may be extended to include many other cascade processes. The prediction was demonstrated by the use of Y346S FNR (denoted FNR*) in conjunction with NADH-dependent malate dehydrogenase (non-decarboxylating) to convert malate into oxaloacetate.^269^

### 5.7 Exploiting the e-Leaf to Investigate Enzyme Inhibitors

**Figure 23:**
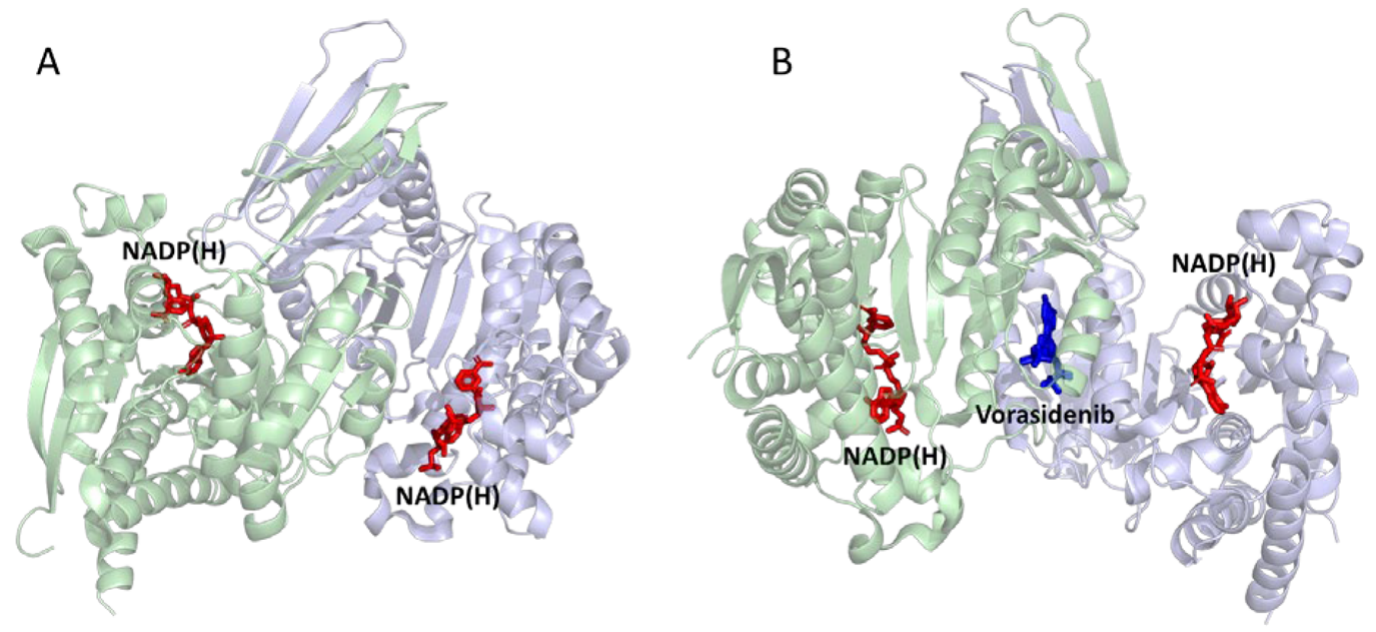
(left) Human isocitrate dehydrogenase 1 (IDH1), showing the dimer structure and one molecule of copurified NADP(H) bound at each monomer active site (red) (PDB: 1T09).^275^ (right) R132H variant of IDH1 showing a single molecule of an allosteric inhibitor (vorasidenib) bound at the dimer interface (blue) (PDB: 6ADG).^277^

The equivalence between electrocatalytic current and rate makes it easy to investigate, in detail, the mechanisms by which bespoke inhibitors, including important drugs, interact and interfere with their enzyme targets. Isocitrate dehydrogenases catalyze the oxidative decarboxylation of the common metabolite d-isocitrate to form 2-oxoglutarate (hereafter abbreviated as 2OG), IDH1 being a cytosolic enzyme that uses NADP^+^ as an oxidant. The structure of IDH1 is shown in Figure 23: it is a homodimer that copurifies and crystallizes with one NADP(H) bound to each monomer^272−276^ (the significance of this observation will be explained in section 5.8).

**Scheme 2:**
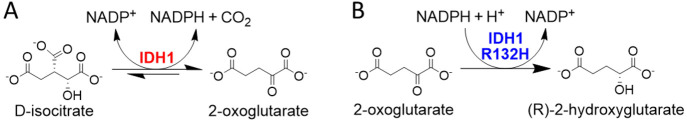
Dominant NADP(H)-Dependent Reactions Catalyzed by (A) Wild-Type IDH1 and (B) IDH1 R132H

The normal reaction catalyzed by IDH1 is shown in Scheme 2 (left) together with the alternative reaction (right) catalyzed by an important variant (IDH1 R132H) that is associated with many different cancers.

#### 5.7.1 Investigating the Mechanism of Drug Binding to Isocitrate Dehydrogenase Variants

Considering one-sixth of all enzymes use NAD(P)(H),^278^ it is not surprising that some (and their associated mutant variants) are implicated in diseases. One notable example was reported in 2009 by Dang et al., who showed that a mutant isocitrate dehydrogenase variant (IDH1 R132H)—already known to be associated with numerous cancers—possessed the ability to perform a new NADP(H)-dependent reaction, the reduction of 2OG to 2-hydroxyglutarate (2HG). This neomorphic activity coincided with a massive decrease in the enzyme’s normal activity (isocitrate oxidation to 2OG and CO_2_) and a buildup of 2HG in cells.^279^ The discovery was extremely significant because it suggested a mechanistic link between metabolic changes induced by a specific enzyme variant and the development of cancer, opening up a new field of research into the enzyme and other IDH cancer-associated variants and ultimately leading to development of drugs to treat acute myeloid leukemia and likely other cancers in the future.^280^ With human IDH1 being implicated in so many different cancers,^281^ understanding the complex, highly dynamic, catalytic mechanisms of both the wild-type enzyme and the cancer-associated variants is of great interest.

**Figure 24:**
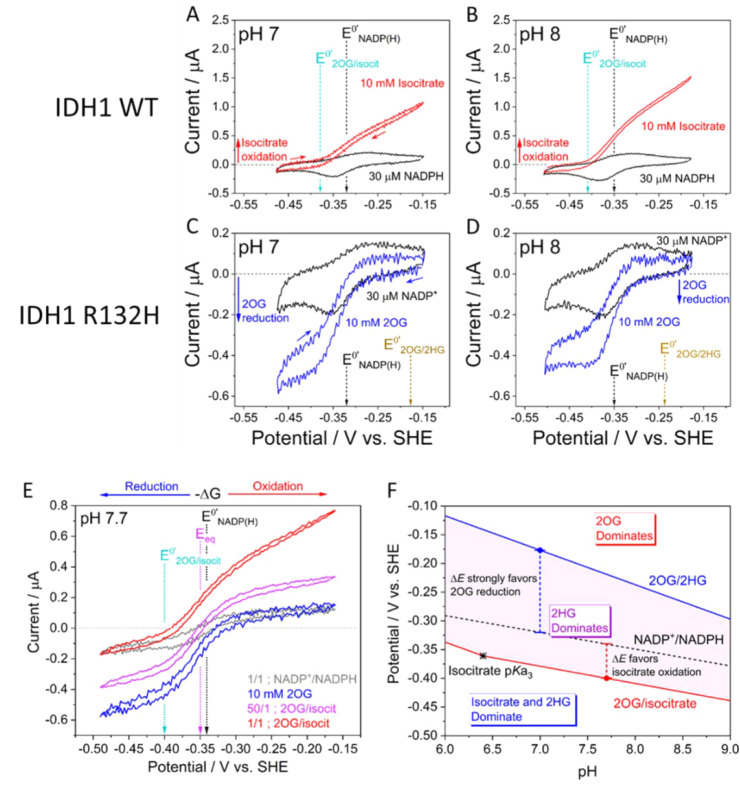
Electrochemistry of nanoconfined wild-type IDH1 and IDH1 R132H. (A, B) Voltammograms for wild-type IDH1 show that it is an efficient catalyst for isocitrate oxidation, with the linear potential dependence of the current indicating that the system is limited by the rate of electron transfer from FNR to the electrode. (C, D) Voltammograms for IDH1 R132H show that it catalyzes its dominant reaction (2OG reduction) much less effectively than the wild-type enzyme oxidizes isocitrate, despite the IDH1 R132H electrode having been loaded with a 4-fold higher IDH1/FNR ratio than the wild-type IDH1 electrode. A sigmoidal catalytic wave-shape indicates that the system is limited by IDH1 R132H turnover, not by electron transfer to FNR. (E, F) Thermodynamics of the reactions catalyzed by wild-type IDH1 and cancer-associated IDH1 variants. (E) Cyclic voltammetry showing reversible wild-type IDH1 catalysis used to determine the isocitrate/2OG, CO_2_ formal potential (*E*^0′^_2OG/isocit_). (F) Pourbaix diagram showing how the 2OG/isocitrate, 2OG/2HG, and NADP^+^/NADPH formal potentials vary with pH. The purple shaded region shows the conditions of potential and pH that promote spontaneous formation of the oncometabolite, 2HG, from isocitrate and 2OG. The 2OG/2HG formal potential was calculated from literature equilibrium data.^283,284^ (Adapted with permission from ref (282). Copyright 2021 American Chemical Society.)

Studying enzymes using the e-Leaf provides the significant benefit of direct insight into *both* the thermodynamics and kinetics of enzyme cascades. Useful interpretation depends, of course, on the fundamental kinetic properties of an enzyme remaining unchanged in a crowded nanoporous environment, but as argued in section 3, such conditions may resemble those in a cell and be more relevant than measurements made in dilute solutions. The activity profiles for IDH1 and IDH1 R132H, the natural reactions of which involve oxidation or reduction, respectively, are readily observed and compared using cyclic voltammetry, as shown in Figure 24A–D.^282^

A much higher loading ratio (IDH to FNR) is needed to observe a sizable current with the variant, consistent with the reduction reaction (2OG to 2HG) catalyzed by IDH1 R132H being much slower than the oxidation catalyzed by the wild-type enzyme (isocitrate to 2OG, CO_2_).^272^ In accordance with their respective activities, the shapes of the cyclic voltammograms (linear potential-dependent for IDH1 vs sigmoidal for IDH1 R132H) are clear indicators for which enzyme (FNR or IDH) is rate limiting.^282^ It is easy to gain direct insight into the thermodynamics underpinning production of the oncometabolite, 2HG, both immediately from 2OG, the substrate of IDH1 R132H, and indirectly from isocitrate, the natural substrate of wild-type IDH. Using the formal reduction potential measured for the isocitrate to 2OG reaction (Figure 24E) and equilibrium data,^283,284^ a Pourbaix diagram could be constructed from which the reactions could be compared over a range of potential and pH (Figure 24F). Interpreting this information, it was clear that IDH1 R132H must facilitate the spontaneous conversion of isocitrate to 2HG in a living cell.

**Figure 25:**
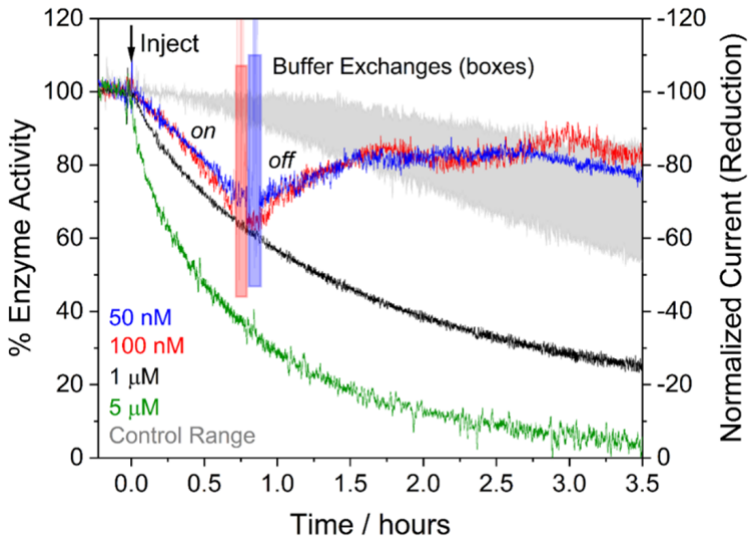
Inhibition kinetics of nanoconfined IDH1 R132H. Time-course experiments showing the effect of different concentrations of the FDA-approved inhibitor, ivosidenib, on the 2OG reduction activity of IDH1 R123H when injected into the buffer solution (at *t* = 0). The increases in current for the blue and red traces (50 and 100 nM, respectively) following live buffer exchanges (with buffer not containing the inhibitor) show that ivosidenib inhibition is reversed in a slow process. The shaded region shows the range for three control experiments where DMSO was injected into the solution without any inhibitor. (Adapted with permission from ref (282). Copyright 2021 American Chemical Society.)

The e-Leaf is ideally suited to study slow binding and release of inhibitors under steady-state conditions because time-dependent changes in rate translate directly into changes in current, and inhibitors may be removed as well as added. In addition, since the enzymes are not distributed in the solution but located only in the electrode nanopores, the total amount of enzyme present is low relative to the amount of inhibitor in the bulk solution, enabling conditions for pseudo-first-order reaction kinetics even if the inhibitor is present only at very low concentration in bulk solution, provided the electrode is rotated to achieve adequate mass transport.^282^Figure 25 shows some results from a study of the mechanism of inhibition of IDH1 R132H by ivosidenib, an FDA-approved IDH1 variant inhibitor. Injections of ivosidenib during 2OG reduction initiate decreases in the steady-state catalytic current. The exponential time-courses are consistent with the binding of a single drug molecule per dimer as indicated in the structure shown in Figure 23, which reveals another drug, vorasidenib, bound at the dimer interface (alternatively, two drug molecules would have to bind at identical rates at each monomer). The kinetics are first order to two half-lives or more, and the extent of reaction decreases as lower levels of ivosidenib are injected. Following a live buffer exchange, in which the reaction buffer was serially diluted with fresh buffer of identical composition *without* inhibitor, the activity of the enzyme is slowly restored as the inhibitor disassociates (Figure 25).^282^

These results indicate how a detailed picture of the transient kinetics of inhibitor binding and dissociation *during turnover* can be developed by determining how the rate constants depend on the concentration of ivosidenib over a range of concentrations of 2OG and Mg^2+^ that covers their likely physiological values.

### 5.8 Localization of Cofactor Recycling

In this closing subsection, we outline experiments that have a more fundamental bearing on the status of nicotinamide cofactors as mobile hydride transfer agents. Conventional dogma has long portrayed NAD(P)(H) existing as a central “pool” within cells and their components, but this may be far from the case—NAD(P)(H) recycling often being a rapid and highly localized process.

#### 5.8.1 Levels and Status of Nicotinamide Cofactors in Cells

Despite the biological importance of nicotinamide cofactors and over 100 years of research,^285^ it has proved difficult to quantify the different nicotinamide species within living cells and their compartments, and relatively few measurements of *in vivo* nicotinamide concentrations have been published. The fluorescence of NADH and NADPH in the 420–480 nm range has been exploited to quantify their levels in biological environments;^285^ however, measuring the “autofluorescence” of living cells does not distinguish between NADH and NADPH, bound or unbound, and gives no information about the concentration of oxidized nicotinamide (NAD^+^ and NADP^+^) present. The latter point is important because NAD(P)^+^/NAD(P)H ratios should reflect local redox status and be central to homeostasis in the living cell and its microenvironments. In contrast to *in vivo* approaches which seek to minimally disrupt normal cellular conditions, *in vitro* methods require cell lysis but yield total concentrations of particular nicotinamide forms. Thus, while *in vivo* measurements of total NAD(P)(H) in different cells range from 0.1 to 0.2 mM,^286,287^*in vitro* estimations are higher, at 0.25–0.9 mM.^288,289^ Moreover, detailed studies show that the majority of intracellular nicotinamide is enzyme-bound,^286,290^ a hypothesis that Theorell and Bonnichsen put forward in 1951;^291^ this may not be surprising given the high concentration of proteins in cells and the number of competing cofactor binding sites that must be present, yet the consequences are far removed from the way that dehydrogenase kinetics have long been studied in the laboratory. The lack of insight into nicotinamide status has important implications for the activity of NAD(P)(H)-dependent enzymes with different *K*_m_ values for NAD(P)(H) and makes dilute solution assays less reliable for predicting enzyme activity *in vivo*. The questions of how tightly these cofactors bind and how their binding strength depends on the catalytic state of the enzyme are relevant for the actions of dehydrogenases under nanoconfinement and in the e-Leaf in particular. Although nicotinamides are usually considered as *exchangeable* cofactors (or cosubstrates), there does exist a class of enzymes that use nicotinamide as a permanently bound prosthetic group to catalyze reactions—the nicotinoproteins.^292−299^ These enzymes represent a limiting case: they bind nicotinamide much more tightly than traditional NAD(P)(H)-dependent enzymes and during a catalytic cycle require the nicotinamide to be reoxidized/reduced while it remains enzyme-bound, analogous to how FAD or FMN cofactors function in enzymes. A permanently bound nicotinamide cofactor is suited to catalyze the hydride exchange required in the dismutation of aldehydes to alcohols and carboxylic acids.

#### 5.8.2 Localized NADP(H) Coupling in the e-Leaf

Early experiments indicated that NADP^+^ and NADPH become concentrated in the ITO pores relative to their concentrations in bulk solution, and that its escape into bulk solution was impeded^235^. Such retention is advantageous for operating cascades as the amount of cofactor required is greatly decreased; accordingly, e-Leaf processes have typically used very low bulk concentrations of NADP(H) in the 5–20 μM range, whereas in most published studies, cofactor concentrations range from 0.1 to 5 mM. As outlined in sections 3, 4, and 5.5, confining the enzymes of a cascade within a closed environment (or pore) increases efficiency by retaining intermediates and preventing the escape of exchangeable cofactors.

**Figure 26:**
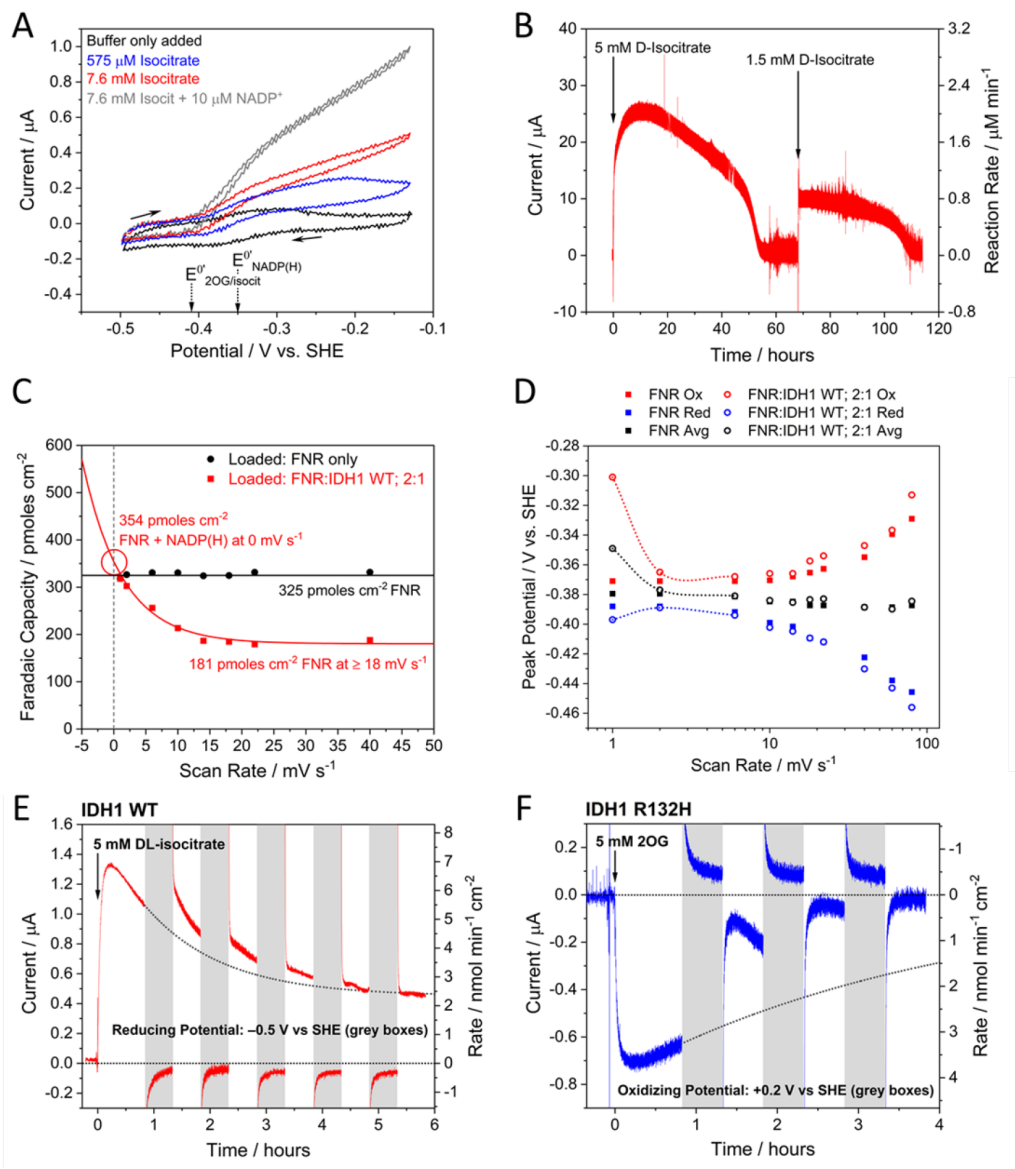
Nanoconfined IDH1 biocatalysis using only copurified, enzyme-bound NADP(H). (A) Cyclic voltammetry (at a stationary electrode) for wild-type IDH1 with isocitrate titrated into solution without any added NADP(H) (except for the gray trace, where 10 μM NADP^+^ was added). (B) Scaled-up experiment (4 cm^2^ ITO on titanium foil electrode) demonstrating conversion of ∼6 mM isocitrate (4 mL solution) to 2OG using only the IDH1 copurified NADP(H). (C) Scan-rate-dependent coverage plot for two electrodes (one coloaded with IDH1 and FNR and the other with FNR only) showing that the presence of nanoconfined NADP(H) (carried into pores by IDH1) can be detected at low scan rates; at high scan rates, the plot reverts to the “FNR-only” signal. (D) Trumpet plot showing the changes in oxidation and reduction peak potentials as a function of scan rate for an electrode loaded with both IDH1 and FNR versus one loaded with FNR only. At low scan rates, the average peak potentials are more positive and are similar to those expected for NADP^+^/NADPH. At high scan rates, the peak potentials mirror those of an electrode loaded only with FNR. (E, F) Oscillating potential switch experiments showing that wild-type IDH1 has a high affinity for both NADP^+^ and NADPH (panel E), whereas IDH1 R132H has a much lower affinity for NADP^+^ compared to NADPH (panel F). Once steady-state catalysis was achieved in each case, the potential was switched back and forth from oxidizing to reducing (+0.2 to −0.5 V) in hour-long cycles to stop catalysis and convert all trapped NADP(H) into either NADP^+^ (oxidizing potential) or NADPH (reducing potential). Only NADP(H) that was tightly held (rebound) by IDH1 or IDH1 R132H would remain trapped in the nanopores, allowing the affinity of each enzyme for particular redox (hydrogenation) states of NADP(H) to be investigated. (Adapted with permission from ref (301). Copyright 2022 National Academy of Science.)

As shown in Figure 23A, human IDH1 copurifies with one molecule of NADP(H) bound at each monomer active site.^272−276,300^ The tight binding of the cofactor to a resting inactive state of the enzyme (which is not reflected in steady-state *K*_M_ determinations) allows each half of the IDH1 molecule to deliver up to a 1:1 stoichiometric quantity of NADP(H) into the pores alongside FNR, without any exogenous NADP(H) being required. Based on a proposed model, only after Mg^2+^ and isocitrate bind to the enzyme to initiate a catalytic cycle is the cofactor released for rapid recycling by a nearby FNR molecule.^301^ The loading of IDH1 with its cofactor cargo resembles advanced drug delivery mechanisms where shuttle particles carry tightly bound cargo molecules to specific locations before they are released when instructed. With a small electrode (held stationary to limit mass transport from bulk solution), low levels of isocitrate are depleted, as shown by the blue CV in Figure 26A. Large amounts of isocitrate are oxidized over >4 days by a scaled up (FNR+IDH1)@ITO/Ti foil electrode system without introducing any additional NADPH, yielding a TTN of 160,000 for NADP(H)(Figure 26B). The “buffer only added” experiment also shown in Figure 26A would normally be expected to represent the reversible two-electron electrochemistry of FNR, similar to that shown in Figure 12A; however, the shape (recorded at 1 mV s^–1^) is distorted because it includes a contribution from the NADP(H) that has been transferred into the electrode by IDH1. Analysis of the reduction and oxidation peak positions (the trumpet plot shown in Figure 26D) and the effective coverage (the integrated “Faradaic capacity” under the peaks) (Figure 26C) as a function of scan rate showed that coupling to NADP(H) begins below 10 mV s^–1^. At higher scan rates, the signal is due only to the reversible electron exchange with the FAD molecule within FNR, whereas below 10 mV s^–1^ the oxidation and reduction peaks shift toward values more representative of NADP(H). The Faradaic capacity extrapolated back to a scan rate of 0 mV s^–1^ indicated that approximately one NADP(H) is undergoing turnover per FNR molecule (i.e., the FNR/NADP(H) ratio in the nanopores is roughly 1/1). It was thus possible to estimate the quantity of IDH1 loaded alongside FNR: the FNR quantity was obtained from the Faradaic capacity at scan rates above 10 mV s^–1^, while the amount of IDH1 was estimated by multiplying the extrapolated NADP(H) component by the fractional occupancy of NADP(H) per IDH1 measured in separate NMR and non-denaturing mass spectrometry experiments on the same IDH1 sample. Taking the quantities obtained alongside assumptions about ITO layer depth and void volume (section 5.2.3), the concentration ranges for the two coloaded enzymes were estimated to be 0.6–1.2 mM for FNR and 0.6–1.3 mM for IDH1. A comparison with the estimates for limiting enzyme concentrations given in Figure 15 indicate that the enzymes must be very crowded; likewise, the higher concentration of FNR that is achieved in the absence of IDH1 also suggests competition for space.^301^

Further experiments were carried out to address the question of whether pausing catalytic turnover results in preferential dissociation of NADP^+^ (under an oxidizing potential) or NADPH (under a reducing potential) (Figure 26E,F). Chronoamperometry experiments in which the potential was periodically switched between oxidizing and reducing values revealed that the 2OG-reducing IDH1 R132H/FNR system loses activity after the first oxidative pause, whereas the isocitrate-oxidizing wild-type IDH1/FNR system is much more stable following a reductive pause.^301^ A useful analogy is found with an “electromagnetic gripper”, a machine that picks up or drops a steel load depending on the electrical command given. Together, the results indicate that wild-type IDH1 retains both NADP^+^ and NADPH in the nanopores (likely through rebinding between catalytic cycles) whereas the R132H variant retains NADPH but releases NADP^+^—a property that may help explain why the neomorphic IDH1 variants preferentially catalyze the 2OG reduction reaction over the wild-type isocitrate oxidation reaction.^301^

Finally, it was possible to estimate the advantage that nanoconfinement provides for cofactor recycling by comparing the concentrations of NADP(H) required to produce the same current in the presence or absence of isocitrate—an enhancement of more than 2 orders of magnitude could be concluded.^301^

## 6 Summary

The e-Leaf combines biocatalysis, electrochemistry, and nanomaterials and provides a platform whereby enzyme cascade catalysis is packaged into a thin-film material. Whereas PFE, outlined in section 2, was previously limited to enzymes able to undergo long-range electron transfer, it now becomes possible to apply the power of electrochemical methods to study a much wider range of enzyme classes that may include transferases (EC 2), hydrolases (EC 3), lyases (EC 4), isomerases (EC 5), and ligases (EC 6). The expansion of the field depends on the manner in which nanoconfinement results in the tightly channelled flow of information along a cascade chain, at one end of which is an FNR/dehydrogenase pair (each belonging to class EC 1). It is this systematic flow of information and the response of components to particular interventions that give rise to the analogy to electronic circuitry mentioned in the abstract, i.e., “cascade-tronics” (it is also 30 years since succinate dehydrogenase was suggested to behave as a tunnel diode^302^). The ideas on catalytic bias outlined for electron-transferring enzymes in conventional PFE can still be applied, with minor modification—the difference of main interest now being the separation between reduction potentials for the nicotinamide cofactor and the reaction being catalyzed. The technology opens up new applications in enzyme research as well as production of high-value chemicals from simple compounds in processes requiring sequential enzyme-catalyzed steps. Catalytic networks might be designed and optimized for such processes by applying AI/machine learning to help identify the most suitable enzymes, drawn from any organism, which can now be combined together in a material.

Regarding the relationship with other recent approaches to study and exploit enzyme catalysis under nanoconfinement that were outlined in sections 3 and 4, a list of advantages and disadvantages can be drawn up. The advantages of the e-Leaf stem from a combination of factors.

1. Being electrochemical, enzyme cascade reactions can be driven and controlled through the electrode potential, and their rates or changes in rate are observed directly, i.e., in real time, as current. Depending on how closely the thermodynamics of the overall reaction and that of the nicotinamide redox reactions coincide, it is also possible to drive a reaction in either direction—the bidirectionality advantage. The transducer FNR has a very high affinity for the ITO pores, and interfacial electron transfer to/from the FAD is very efficient: artificial mediators are thus completely avoided.
2. All enzymes are loaded into a nanoporous thin layer of micron depth: they are naturally confined in the randomly formed tunnels in the material, and their concentrations may approach the maximum possible values based upon their size. Their natural entrapment in the porous network, which must stem at least partly from their large size, makes it possible to channel extended cascades with minimal release of intermediates and extremely efficient use of exchangeable cofactors such as NAD(P)(H) or ATP. Total turnover numbers (TTNs) of these cofactors may reach very high levels, enabling them to be used economically in biocatalytic synthesis.
3. As with PFE, the catalytic wave-shape observed in CV experiments is very informative, and it reports on the factors controlling the performance of the cascade. As seen from some results described above, the wave-shape may be peak-like (local depletion), sigmoidal (steady state rate controlled by enzyme activity), or linear (rate controlled by electron transfer with FNR).
4. The materials and experimental set up are simple and inexpensive.

Likewise, disadvantages of the e-Leaf are as follows.

1. Being heterogeneous, the number of catalytic units is limited by the surface area of the electrode that is available to reactants, unlike a suspension in bulk solution. However, the electrode size can be scaled up to compensate for this shortcoming—Ti foil, for example, is coated on both sides, and large areas can be fabricated to fit inside electrochemical reactors.
2. Apart from FNR, active enzyme loadings may be difficult to quantitate directly, and specific interenzyme distances are not defined.

On balance, the spatial organization of the enzymes in the ITO film will be random unless procedures are devised to create multienzyme complexes akin to metabolons. Given the high concentration of trapped enzymes in the pores, the question of specific interenzyme distances may not be so relevant. The random yet homogeneous arrangement of all enzymes otherwise represents an advantage as all enzyme partner combinations are likely to be present. The performance is easily optimized by varying the amount and ratios of the enzymes that are loaded, and interpreting the corresponding CV shape and amplitude. The catalytic bias that determines bidirectionality is now related to the difference in reduction potential between that of NADP^+^/NADPH and the reaction being catalyzed. We can thus correlate the electrocatalytic voltammograms of electron-transferring enzymes shown in Figure 5 with those for ketone/alcohol and 2OG-CO_2_/isocitrate shown in Figures 16 and 24, respectively. Note that although FNR is more reducing than NADPH, it is the latter that appears to parallel the electrochemical control site in ET enzymes.

These views raise more fundamental questions. A nanoporous environment in which enzymes are both crowded and trapped is very different to the dilute solution environment that has long been assumed in enzyme kinetics. Diffusion of reactants and products between enzymes will be fast and the escape of intermediates restricted, the importance of which was stressed in section 4. The crowding of enzymes means that assumptions commonly adopted for enzyme kinetics break down; for example, the condition [E] ≪ [S] may no longer apply. Enzymes behave differently under nanoconfinement in other ways, examples being the rapid racemization of enantiomeric secondary alcohols (section 5.4) and the entrapment of AMP that hinders the activation of CAR (section 5.5). Beyond displacing a large amount of solvent water, it remains unclear, at the detailed level, why enzymes should “prefer” to become concentrated and crowded in the electrode pores rather than exist as free molecules in dilute solution.

Cascade catalysis in the e-Leaf is enhanced by many of the same attributes recognized to be important in living cells. In the highly porous electrode, the cascade enzymes are crowded together in cavities where they remain nanoconfined under similar conditions to the cell. Moreover, just as in nature where the pathways of metabolism are spatially confined inside organelles or in cytoplasmic zones, in the e-Leaf, the enzymes function in this crowded pore as a “team”, with intermediates and exchangeable cofactors also retained, since, rather than diffusing out of the pore, they are more likely to encounter another enzyme in the team, akin to cluster channelling, as defined by Benkovic.^130^ Moreover, just as nature compensates for the very slow rate of RuBisCO, by packing it into the carboxysome at ∼7 times the amount of carbonic anhydrase, the alteration of enzyme ratios to compensate for inherently slow enzymes is also possible in the e-Leaf (see section 5.5, Extended Cascades).

Parallels can also be drawn between the e-Leaf and nature’s temporal control over enzyme cascades. In electrochemistry, the driving force is delivered via the electrode potential while timing is achieved via the scan rate (cyclic voltammetry) or directly as in chronoamperometry. Therefore, just as biology controls catalytic pathways by switching cascades off and on (for example, by differential transcription) and by controlling their flux (for example, by allosteric regulation), in the e-Leaf, cascade activity can also be switched off and on, paused, or reversed in direction. The ability to rotate the electrode at high speed provides another way to control activity, by bringing reactants to the electrode or assisting their release. These factors, along with the ability to observe and measure, are the basis of the “dashboard” analogy introduced in section 5.3. Through robust and energizable nanoconfinement, it may now become much easier to exploit enzymes interactively and navigate complex catalytic networks.
